# Enzymatic and proteomic exploration into the inhibitory activities of lemongrass and lemon essential oils against *Botrytis cinerea* (causative pathogen of gray mold)

**DOI:** 10.3389/fmicb.2022.1101539

**Published:** 2023-01-18

**Authors:** Itumeleng E. Kgang, Ashwil Klein, Gadija G. Mohamed, Patricia M. K. Mathabe, Zinash A. Belay, Oluwafemi James Caleb

**Affiliations:** ^1^Department of Biotechnology, University of the Western Cape, Western Cape, South Africa; ^2^Post-Harvest and Agro-Processing Technologies (PHATs), Agricultural Research Council (ARC) Infruitec-Nietvoorbij, Stellenbosch, South Africa; ^3^School of Agriculture, Food & the Environment, Royal Agricultural University, Cirencester, United Kingdom; ^4^Department of Food Science, Faculty of AgriSciences, Stellenbosch University, Stellenbosch, South Africa; ^5^African Institute for Postharvest Technology, Faculty of AgriSciences, Stellenbosch University, Stellenbosch, South Africa

**Keywords:** necrotrophic pathogens, ascorbate peroxidase, superoxide dismutase, malondialdehyde, bioassays

## Abstract

**Introduction:**

Essential oils (EOs) have been demonstrated as efficacious against *B. cinerea*. However, the underpinning enzymatic and proteomic mechanism for these inhibitory effects is not entirely clear.

**Methods:**

Thus, this study examined the effects of lemon (Le) and lemongrass (Lg) EOs (individually and in combination) against *B. cinerea* based on enzymatic and proteomic analyses. Proteomics data are available via ProteomeXchange with identifier PXD038894.

**Results and discussion:**

Both EOs (individually and in combination) displayed abilities to induce scavenging as observed with the reduction of H2O2. Measured malondialdehyde (MDA) and superoxide dismutase (SOD) activity were increased in all EOs treated *B. cinerea* mycelia compared to the control. Ascorbate peroxidase (APX) activity was highest in Lg treated *B. cinerea* (206% increase), followed by combined (Le + Lg) treatment with 73% compared to the untreated control. Based on GC-MS analysis, the number of volatile compounds identified in lemon and lemongrass EOs were 7 and 10, respectively. Major chemical constituent of lemon EO was d-limonene (71%), while lemongrass EO was a-citral (50.1%). Based on the interrogated LC-MS data, 42 distinct proteins were identified, and 13 of these proteins were unique with 1, 8, and 4 found in Le-, Lg-, and (Le + Lg) EOs treated *B. cinerea*, respectively, and none in control. Overall, 72% of identified proteins were localized within cellular anatomical entity, and 28% in protein-complexes. Proteins involved in translation initiation, antioxidant activity, protein macromolecule adaptor activity and microtubule motor activity were only identified in the Lg and (Le + Lg) EOs treated *B. cinerea* mycelia, which was consistent with their APX activities.

## Introduction

Among the necrotrophic pathogens, *Botrytis cinerea* is one of the most researched fungal species due to its broad host range, causing fungal diseases in over 500 plant species. *B*. *cinerea* is known to cause major agricultural losses in crops that are of economic importance (Cheung et al., 2020). The postharvest growth and spread of *B*. *cinerea* are managed by numerous methods such as the use of chemical fungicides, sulphur dioxide (Kgang et al., 2022), modified and/or controlled atmosphere storage (Artés-Hernández et al., 2004, 2006). However, chemical compounds used as antimicrobial agents to manage *B*. *cinerea* faced major resistance due to growing concerns of environmental pollution and possible adverse impact on public health (Brink et al., 2016; Kgang et al., 2022).

Therefore, safe alternatives are needed for the postharvest management of pathogenic fungi. Application of natural plant extracts like essential oils (EOs) has been considered a viable method for controlling fruit disease causing pathogens. Several research have shown that EOs have antimicrobial (Yang et al., 2020a,b), antiviral (Brochot et al., 2017), and antifungal (Gakuubi et al., 2017; Powers et al., 2018; de Oliveira Filho et al., 2021; OuYang et al., 2021; Kgang et al., 2022) properties against diverse range of microorganisms. Yang et al. (2021) recently provided an extensive review on the mechanism of action of EOs against microorganisms. Based on available literature, EOs can initiate the biosynthesis of reactive oxygen species (ROS), which can contribute to H_2_O_2_ cytotoxicity in pathogenic fungi causing damage to various cellular components and ultimately cell death (Mani-López et al., 2021).

The ROS molecules are made up of radical and non-radical oxygen species that are created by the partial reduction of oxygen. This includes nitric oxide (NO), superoxide anion (O_2_^–^), hydroxyl radicals (OH⋅), and hydrogen peroxide (H_2_O_2_) (Mani-López et al., 2021). These molecules have important roles at cellular level in signaling and homeostasis (Dwyer et al., 2009). In addition, these ROS degrades cellular components such as DNA, RNA, protein, and lipids. It damages cell membrane stability, which could result in suppression of germination of spores/conidia, and inhibition of pathogen growth (Liu et al., 2010; Kalagatur et al., 2018). To date, no reports have elucidated on the underpinning mechanisms of the lemon and lemongrass EOs and their mixtures on ROS metabolism and antioxidant activity of *B*. *cinerea*. Similarly, the proteomic changes in *B*. *cinerea* exposed to lemon and lemongrass EOs remains unknown and have not been associated with specific proteins.

To comprehend the enormous complexity of fungal biochemistry, proteomics has become an essential component in understanding fungal pathogenicity, virulence, and plant-fungus interactions (Mathabe et al., 2020; Belay and Caleb, 2022). Proteomics is an emerging technology adapted to study the cumulative shifts in protein abundance (regulations) in response to environmental signals and their interactions with other proteins and molecular entities (Mathabe et al., 2020). Most of the proteomic research conducted on *B*. *cinerea* focused on investigating the proteome of the mycelium via gel electrophoresis (using two-dimensional, 2-DE gel) in combination with MALDI-TOF MS/MS (Fernández-Acero et al., 2006, 2009, 2010). Although 2-DE is widely applied in proteomics for their robustness and simplicity, it has many limitations. These include poor reproducibility of hydrophobic, highly basic, and highly acidic proteins (Ong and Pandey, 2001).

Furthermore, this approach has been said to underrepresent low abundant proteins and proteins with extreme physico-chemical features (such as isoelectric point, size, transmembrane domains) (Bianco and Perrotta, 2015). First application of 2-DE gel to investigate filamentous fungus was reported by Grinyer et al. (2004). Using a combination of 2-DE with MALDI-TOF, they were able to map hundreds of proteins on 2-D gels from *Trichoderma harzianum*. However, from the total cell protein reference map, the authors identified only 25 proteins (Grinyer et al., 2004). Due to these limitations in 2-DE protein profiling, the scientific community has shifted its focus away from gel-based proteomics and toward alternative proteomic techniques known as gel-free proteomics, based on protein separation by liquid chromatography (LC).

Recent study by Kgang et al. (2022) characterized the secondary metabolites for lemon, lemongrass, and peppermint EOs, and reported on their antifungal efficacy individually and in combination against *B*. *cinerea* and *Penicillium expansum*. The authors were able to establish minimum inhibitory concentrations of 5 mg/L for Lg EO against both mycelial growth and spore germination of *B*. *cinerea*. However, the underpinning mechanisms for the inhibitory effects were not directly accounted for. Overall, there is limited understanding on how fungal pathogens response to the induced stress after the application of EOs at proteomics level. It is therefore imperative to understand its mechanism of action in addition to the EOs mixture. Thus, the aim of this study was to evaluate the effects of lemon and lemongrass EOs on induced ROS oxidative damage in *B. cinerea* and monitor the changes in activities of ROS scavenging antioxidant associated enzymes, based on enzyme assays and the integration of both gel-based and -free proteomics coupled with LC-MS/MS analysis.

## Material and methods

### Fungal pathogen and conidial suspension

*Botrytis cinerea* strain PPRI 7338 was acquired from the Agricultural Research Council (ARC) - Plant Health and Protection Institute in Pretoria, Mycology Division, South Africa. Strain PPRI 7338 was isolated from infected apples. Potato dextrose agar (PDA, NutriSelect^®^Plus, Merck (Pty) Ltd., Johannesburg, South Africa) was used to culture the inoculum of *B. cinerea* PPRI 7338, and the plates were stored at 20°C for 5 days. Conidial stock suspensions were prepared and conserved as reported by Vallejo et al. (2002) with few modifications. Six mycelial plugs (0.432 cm^2^) from 5-day-old *B. cinerea* cultures maintained on PDA media were transferred to flasks (250 mL) with 100 mL of minimal salts medium (Supplementary Table 1) augmented with 1% Carboxymethylcellulose (CMC).

Conidial suspensions of *B. cinerea* were cultivated for 7 days at 20°C and agitated at 180 rpm using Orbital Shaker (OrbiShake, Labotec, Cape Town, South Africa). *B. cinerea* cultures supplemented with lemon and lemongrass essential oils individually (20 mg/L) and in combination (1:1, 20 mg/L) that were harvested at day 7 were used for enzymatic and proteomic analysis. Using Eppendorf microcentrifuges (5425 R, Stevenage, UK), treated and non-treated *B. cinerea* cultures were centrifuged for 10 min at 13 000 x g, and the mycelia stored at −80°C before other downstream analysis were conducted. This experiment was independently replicated in triplicate and three times (*n* = 9).

### Mycelium preparation for biochemical analysis

Mycelia harvested from treated and non-treated *B. cinerea* were grounded using liquid nitrogen into fine particle size. Mycelia (0.2 g) from each treatment was homogenized in 1 mL of 6% (w/v) trichloroacetic acid (TCA) for analysis of H_2_O_2_ content, and lipid peroxidation. For the measurement and detection of superoxide dismutase (SOD) and ascorbate peroxidase (APX) enzymatic activities, 1 mL of homogenizing PVP extraction buffer (40 mM K_2_HPO_4_ at pH 7.4; 1 mM EDTA; 5% PVP MW = 40,000; 5% glycerol in distilled H_2_O). Concentrations of protein for each sample was quantified using the reducing agent and detergent compatible (*RC DC*™) Protein Assay Kit II (Bio-Rad Laboratories Ltd., Rosebank, Johannesburg, South Africa).

### Extraction of total soluble protein from *B. cinerea* mycelia

Freshly harvested mycelia (0.1 g) from each treatment were ground to a fine powder in liquid nitrogen and 0.5 g PVPP. Total soluble proteins were isolated using a method by Röhrig et al. (2006) with slight modifications previously described by Wang et al. (2006). Mycelia powder was homogenized in 10% (w/v) TCA/Acetone and centrifuged at 13 000 x g for 6 min. The obtained pellets were washed with 80% (v/v) methanol containing 0.1 M ammonium acetate, after the supernatants were discarded. This was followed by acetone wash with 80% (v/v). The pellets were allowed to air dry at room temperature for an hour and re-suspended in a 1:1 ratio of phenol and SDS buffer (Supplementary Table 1).

The re-suspended pellets were placed on ice for 6 min, and centrifuged at 13,000 x g for 6 min. The upper phenol phase of each sample was transferred to sterile 2 mL Eppendorf tubes and filled with the 80% (v/v) methanol containing 0.1 M ammonium acetate to allow for protein precipitation overnight at −20°C. The precipitates were centrifuged at 13,000 x g for 6 min and the supernatant discarded. The pellets were washed with 100% (v/v) methanol followed by an additional wash with 80% (v/v) acetone. Pellets were air-dried at room temperature for 1 h and re-suspended in IEF buffer (Supplementary Table 1) for One dimensional polyacrylamide gel electrophoresis (1 SDS-PAGE) analysis.

### Gas chromatography-mass spectrometry analysis

Cold-pressed extracts of lemon (*Citrus limon*, 1001714241), lemongrass (*Cymbopogon citratus*, 101400274) essential oils were obtained from Sigma-Adrich (St. Louis, MO, USA). The EOs were kept refrigerated (4°C) prior to gas chromatography-mass spectrometry (GC–MS) analysis. Each EO was diluted separately in hexane (1:10,000), and 1 μL of the mixture was thereafter injected into the column with a split ratio of 10:1. Chemical constituents of each EO were determined using Agilent 7890A gas chromatography (GC) system coupled with Agilent 5975C mass selective detector MSD (Agilent, Palo Alto, CA) and both equipped with a HP-5MS column (30 m × 0.25 mm i.d., 0.5 μm film thickness).

Helium at a constant flow rate of 1.3 mL/min was the carrier gas used in this measurement. Oven condition was set as follows: 50°C for 3 min, then ramped up to 80°C at the rate of 4°C/min for 3.5 min; and, finally at a rate of 10°C/min temperature was ramped up to 250°C, and held for 6 min. Mass spectra were analyzed in the scan mode over the range of 35 to 600 m/z. Compounds were identified qualitatively by their retention times (RT), retention index (RI) values and mass spectra names obtained from NIST MS library search and only match greater than 90% were accepted. Chemical constituents were identified based on individual external standards and the percentage composition determined under same chromatographic conditions mentioned above. All measurements were conducted in triplicate (*n* = 3).

### Measurement of H_2_O_2_ content

The H_2_O_2_ content of the mycelia was measured as described by Velikova et al. (2000). The reaction mixture consisted of 50 μL TCA extract, 5 mM dipotassium phosphate (K_2_HPO_4_, pH 5.0) and 0.5 M potassium iodide in a final volume of 200 μL. Sample reactions were incubated in the dark at room temperature (25°C) for 20 min. Thereafter, absorbance values were measured using FLUOstar Omega UV-visible spectrophotometer (BMG LabTech GmbH, Ortenberg, Germany) at 390 nm. Using H_2_O_2_ standard curve approach based on known concentrations at absorbance (A390 nm), the *B. cinerea* mycelia H_2_O_2_ content of was calculated and expressed as nmol g^–1^ fresh weight (FW) basis.

### Determination of malondialdehyde content

Malondialdehyde (MDA) is a known biomarker for oxidative stress and end-product of lipid peroxidation (reflective as MDA content). With slight modifications to the thiobarbituric acid (TBA) method reported by Egbichi et al. (2013), MDA content of each sample was measured. For this study, TBA extract (200 μL) was added to 400 μL of cold 20% (w/v) TCA. The mixture was homogenized and centrifuged under refrigerated condition (4°C) at 12,000 rpm for 30 min using Eppendorf microcentrifuges (5425 R, Stevenage, UK). An aliquot portion of the supernatant (100 μL) was mixed with 400 μL of 0.5% TBA (prepared in 20% TCA). This assay was then incubated in a water bath at 95°C for 20 min, thereafter, the reaction was cooled on ice for 5 min, and further centrifuged under refrigerated condition (4°C) at 12,000 rpm for 5 min. To correct for non-specific turbidity in the samples, absorbance of the supernatant was measured at 532 nm and 600 nm. The MDA content was calculated by using an extinction coefficient (ε = 155 mM^–1^cm^–1^) and reported as nmol g^–1^ FW.

### Measurement of ascorbate peroxidase enzyme activity

Ascorbate peroxidase (APX) activity was evaluated based on a modified approach reported by Asada (1992). Each reaction (with final volume of 200 μL) was incorporated with enzyme extract (10 μL) and topped-up with APX reaction buffer (180 μL) (Supplementary Table 1). The reaction was initiated with the addition of 10 μL H_2_O_2_ and the enzymatic activity was measured at 290 nm absorbance. To calculated APX activity, the extinction coefficient (ε = 2.8 mM^–1^ cm^–1^) was use, and results presented as mmol μg^–1^.

### Measurement of superoxide dismutase enzymatic activity

To quantify superoxide dismutase activity, a modified method by Beyer and Fridovich (1987) was used. The reaction of the final mixture volume of 200 μL; containing enzyme extract (10 μL) and SOD (190 μL) reaction buffer (Supplementary Table 1) was initiated by adding riboflavin (2 μM), which was followed by exposure to light for 20 min or the observation of a change in color. Changes in the mixture complex by the addition of riboflavin was measured via absorbance readings at 560 nm. Under standard assay conditions, one unit of SOD activity represents the volume of enzyme required to reduce 50% of NBT to blue formazan.

### 1 SDS-PAGE analysis

A fraction from each protein pellet (10 μg) was denatured and fragmented using 12% SDS-PAGE electrophoresis gel ran for 90 min at 120 V. At the end of the electrophoresis run, the gel was stained for 30 min using Coomassie Brilliant Blue G-250, thereafter, de-stained for 2 h using 10% glacial acetic acid and 1% glycerol. The fractioned/fragmented protein bands on the gels were viewed and captured using the ENDUROTM GDS Gel Documentation System (Labnet International, Edison, NJ).

### Protein pellet solubilization for LC-MS/MS analysis

The left-over fraction of protein pellets was solubilized in solution A (100 mM Tris, 1% TritonX-100 and 4 M guanidine hydrochloride) followed by three cycles of 1-min sonication. After sonication, solution A was decanted and replaced with 1% formic acid (FA) in a new sterile tube (2 mL), and a one-minute sonication cycle was repeated three times. At the end of the repeated 1-min sonication step, FA was decanted and combined with solution A to perform a methanol-chloroform liquid-liquid analysis. Mixture of solution A-FA was blended with methanol (4 volumes). To the solution A-FA-methanol mixture was thoroughly mixed with 1 volume of chloroform. To facilitate partitioning of the mixture, three volumes of water were added, and the solution was centrifuged at 13,000 x g for 5 min. Four volumes of methanol were added after the upper phase of the solution A-FA-methanol-chloroform mixture was discarded. The reaction mixture was centrifuged at 13,000 x g for 5 min and the methanol-chloroform phase separated from the solution. The protein pellets were air-dried at room temperature and re-constituted in 50 mM Tris buffer containing 2% SDS and 4 M urea.

### On-bead digest

Solubilized proteins were re-constituted in triethyl ammonium bicarbonate (TEAB, 50 mM; Fluka™, Honeywell International Inc., Charlotte, North Carolina, US), followed by the addition of triscarboxyethyl phosphine (TCEP, 5 mM; Fluka™, Honeywell International Inc., Charlotte, North Carolina, US) and 100 mM TEAB. This mixture was stored under agitation for 1 h at room temperature. Cystein residues were thiomethylated for 30 min at room temperature, using 20 mM S-Methyl methanethiosulfonate (Sigma Aldrich, Johannesburg, South Africa) in 50 mM TEAB. After thiomethylation two-fold dilution of the samples was conducted using binding buffer (100 mM Ammonium acetate, 30% acetonitrile, pH 4.5).

According to manufacturer’s instructions the protein solution was added to MagResyn HILIC magnetic particles (Resyn Biosciences (Pty), Ltd., Gauteng, South Africa), and the mixture was incubated at 4°C overnight. After overnight incubation and binding, supernatant from the mixture was decanted and the magnetic particles were washed with washing buffer (100 mM Ammonium acetate, 15% acetonitrile, pH 4.5) twice. The washed magnetic particles were added into TEAB (100 mM) containing trypsin (New England Biolabs^®^, Ipswish, UK) to a final ratio of 1:20 and incubated for 4 h at 37°C. At the end of the incubation period, peptides were extracted using water (50 μL) once followed by 50% acetonitrile (ACN). Samples obtained were dried down and re-constituted with 2% ACN:water and 0.1% FA (30 μL). Residue of the reagents used in the digestion step were removed using an in-house manufactured C_18_ stage tip (Empore Octadecyl C_18_ extraction discs; Supelco). The samples were loaded onto the stage tip after activating the C_18_ membrane with methanol (30 μL) and equilibrated with 30 μL of 2% ACN: water and 0.05% trifluoroacetic acid (TFA). Before eluting with 30 μL 50% ACN:water and 0.05% TFA, the bound sample was washed with 30 μL of 2% ACN: water and 0.1% TFA. The eluate was dried and the dried up peptides were dissolved in 2% ACN:water; 0.1% FA for LC-MS analysis.

### Peptide fractionation and detection using LC-MS/MS analysis

To fractionate the peptides before mass spectrometry analysis, a high-pressure liquid chromatography system running at dionex nano-flow rate was used. The procedure for LC-MS/MS analysis was adapted from Yasmeen et al. (2016). Liquid chromatography was performed using a Thermo Scientific Ultimate 3000 RSLC equipped with a 5 mm x 300 μm C_18_ trap column (Thermo Scientific, USA) and an Asentic Express 15 cm x 75 μm of 2.7 μm size C_18_ analytical column (Supelco). The solvent system used for loading were: 2% ACN:water; 0.1% FA; Solvent A: 2% ACN:water; 0.1% FA and Solvent B: 100% ACN: water. Using the loading solvent at a flow rate of 10 μL/min, samples were loaded onto the trap columns via a controlled autosampler set at 7°C. Loading was performed for 4 min before the sample was eluted onto the analytical column, and the flow rate was maintained at 350 nl/min. The following gradient were generated: 5-35% solvent B over 60 min; 35 - 50% solvent B from 60 to 80 min using Chromeleon non-linear gradient. Chromatography was performed at 40°C and the outflow delivered to the mass spectrometer through a stainless-steel nano-bore emitter.

### Liquid chromatography mass spectrometry analysis

Thermo Scientific Fusion mass spectrometer equipped with a Nanospray Flex ionization source was used for detection, and data were collected in a positive mode with spray voltage set to 1.8 kV and ion transfer capillary set to 280°C. Polysiloxane ions at m/z = 445.12003 and 371.10024 were used as internal calibration. MS1 scans were performed using the orbitrap detector set at 60,000 resolutions over the scan range 350-1,650 with AGC target at 5 E4 and maximum injection time of 40 min. Data was acquired in profile mode. MS2 acquisitions were performed using monoisotopic precursor selection for ion with charges + 2 to + 7 with error tolerance set to ± 10 ppm. Precursor ions were excluded from fragmentation once for a period of 60 s. Precursor ions were selected using the quadrupole mass analyzer at higher energy dissociation (HCD) mode with the HCD set to 30%. Fragment ions were detected in the orbitrap mass analyzer set to 15,000 resolutions. The AGC target and the maximum injection time were set to 5 E4 and 30 min, respectively. The data were acquired in centroid mode. The MS proteomics data have been deposited to the ProteomeXchange Consortium via the PRIDE (Perez-Riverol et al., 2022) partner repository with the dataset identifier PXD038894 and 10.6019/PXD038894.

### Quality control and protein quantification

MS-generated files were transferred into Proteome Discoverer software (vr. 1.4, Thermo Scientific, USA). The files were processed using both Sequest and Amanda algorithms. Database interrogation was performed against a concatenated database created using the Uniprot *Botrytis* database concatenated with the cRAP contaminant protein database. Two missed cleavages were set as the allowed semi-tryptic cleavage, while the precursor mass tolerance and fragment mass tolerance was set to 0.05 Da and 10 ppm, respectively. Deamidation, oxidation, and acetylation of protein N-terminal was allowed as dynamic modifications and thiomethylation of C as static modification. Peptide validation was performed using the Target-Decoy PSM validator node set to search against a decoy database with strict FDR 1% and delta Cn of 0.1. Obtained results were transferred to Scaffold 1.4.4 and identified peptides validated using the X!Tandem, peptide and protein prophet search algorithm included in Scaffold. Peptide and Protein Prophet algorithms were used for peptide and protein validation, and the quantitation of protein was performed using Fischer’s Exact Test on the paired data with the Benjamini-Hochberg correction applied. Identified proteins were only considered acceptable if they were confirmed at greater than 95% probability and contained at least two unique identified peptides.

### Statistical analysis

Experimental design used in this study was full factorial. Besides VOC analysis, all assays and proteomics experiment were independently replicated in triplicate and three times (*n* = 9). All statistical analyses for the enzymatic assays were performed using statistical software SAS (vr. 9.2, SAS Institute Inc., Cary, NC, USA). To test for normality, Shapiro-Wilk test was performed. Data obtained were analyzed using One-Way analysis of variance (ANOVA) at p ≤ 0.05, and mean values were tested according to Tukey’s multiple comparison test at p ≤ 0.05, with F-values indicated significant at p ≤ 0.01. An interactive tool, Venny 2.1.0, BioinfoGP was used for comparing lists of Venn’s diagrams (Oliveros, 2007-2015).

## Results and discussion

### Volatile compounds of lemon and lemongrass EOs

Based on GC–MS analysis, 17 volatile compounds were identified across the selected EOs. Lemon (Le) and lemongrass (Lg) EOs consisting of 7 and 10, respectively (Table 1). The major chemical constituent for Lg EO includes α-citral (50.1%), β-citral (35%), geraniol (4%), and geranyl acetate (3%), while other components (camphene, δ-limonene, linalool, α-terpineol, caryophyllene, and γ-cadinene) were 1% each. In contrast, the chemical constituents of Le EO were δ-limonene (71%), β-pinene (16%), and γ-terpinene (7%), with monoterpene hydrocarbons being the most abundance chemical class with 97% and oxygenated monoterpenes with 1.8%. Cumulatively for Lg EO, the oxygenated monoterpenes were the most abundant class (94%), while both monoterpene hydrocarbons and sesquiterpene hydrocarbon were 2%, respectively (Table 1).

**TABLE 1:** Relative peaks (%) of major volatile composition of selected essential oils from GC-FID.

EO composition	RT (min)	RI^a^	RI Lit ^b^	Relative abundance (%)		CAS no.
				Lemon	Lemongrass	
α-Pinene	1.282	939	940	2	_	80-56-8
β-Pinene	1.615-1.618	978	963	16	_	127-91-3
Camphene	1.832	955	954	_	1	79-92-5
β-Myrcene	1.832	993	992	1	_	123-35-3
δ-limonene	2.255	1,029	1,018	71	1	138-86-3
γ-terpinene	2.722	1,063	1,062	7	_	99-85-4
Linalool	3.544	1,101	1,100	_	1	78-70-6
α-terpineol	5.254	1,193	1,241	_	1	98-55-5
β-citral (neral)	6.462-6.516	1,244	1,235	0.8	35	106-26-3
Geraniol	6.936	1254	1,260	_	4	106-24-1
α-citral (geranial)	7.190-7.230	1,272	1,254	1	50.1	5392-40-5
β-Caryophyllene (*E*)	7.670	1,428	1,420	_	1	87-44-5
Geranyl acetate	10.110	1,385	1,414	_	3	105-87-3
γ-cadinene	11.963	1,521	1,496	_	1	39029-41-9
Total No.				7	10	
Total relative peak%				98.8	98.1	
*Monoterpene hydrocarbons*				97	2	
*Oxygenated monoterpenes*				1.8	94.1	
*Sesquiterpene hydrocarbon*				0	2	
*Miscellaneous compounds*				0	0	

^a^Determined retention indices (RI) for n-alkanes on HP-5MS capillary column.

^b^Literature sources: Kgang et al., 2022, and NIST.

Most abundant compounds for Le and Lg EOs in this study differ from those reported in other related studies (Table 1). For example, Lg EO was associated with the abundance of geranial, neral, geraniol, myrcene, and linalool (Munhuweyi et al., 2017; Moutassem et al., 2019). The discrepancies in relative percentage composition of VOCs in EOs could be associated with variations in the plant material parts extracted, the method of extraction and harvested location or agro-climatic regions (Kgang et al., 2022). Similarly, the composition of EOs identified in this study could play a crucial role in their antifungal efficacy (Shah and Mehta, 2018).

### Effects of EOs on hydrogen peroxide content

**FIGURE 1:**
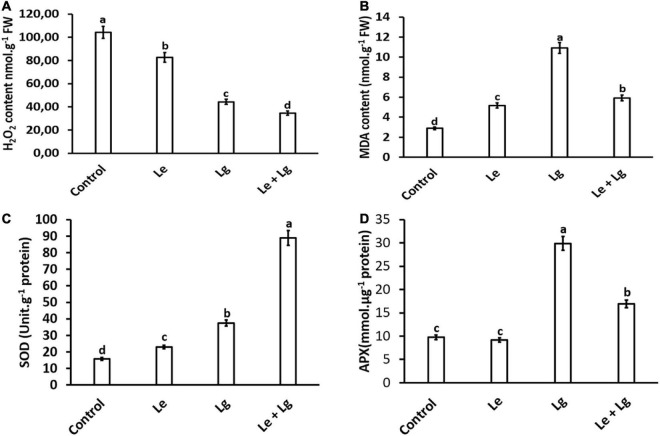
Effect of essential oil singly and in combination on: **(A)** H_2_O_2_ content – induced oxidative stress, **(B)** MDA content – lipid peroxidation, **(C)** SOD activity, and **(D)** APX enzyme activity in *B. cinerea* mycelia after 7-day-incubation. Values represent mean (*n* = 9) ± standard error (S.E.). Various alphabets on the graphs indicate that the average mean values of different treatments are significantly different according to Tukey’s multiple comparison test at *p* ≤ 0.05 (Control, Le – Lemon, Lg – Lemongrass, and Le + Lg – combined effect of lemon and lemongrass).

Hydrogen peroxide is an important biomarker for the membrane lipids peroxidation, and it has been demonstrated to have a direct antifungal effect (Qin et al., 2011; Wu et al., 2022). Results showed that exposure of *B. cinerea* to Le, Lg and Le + Lg EOs significantly reduced H_2_O_2_ content in the fungal mycelia (*p* < 0.005) at the end incubation day 7 (Figure 1A). The reduction in H_2_O_2_ content was most pronounced in the binary mixture involving the two essential oils (Le + Lg). In comparison to the untreated (control), H_2_O_2_ content was reduced by 21%, 58% and 67% under Le, Lg and (Le + Lg) combined treatments, respectively (Figure 1A).

The observation from this study is consistent with literature on the response of *B. cinerea* to EOs and other botanical fungicides (Yang et al., 2020a; Wu et al., 2022). For instance, Yang et al. (2020a) demonstrated that treatment of *B. cinerea* with cembratrien-diols induced oxidative stress, which resulted in injured membrane system with a significant reduction of H_2_O_2_ content in the mycelia. Similarly, H_2_O_2_ content was found to be significantly lower in *B. cinerea* mycelia exposed to juniper EO compared to the untreated control during 12 h incubation period (Wu et al., 2022). The balance between reactive oxygen species (ROS) production and scavenging capacity is crucial for any typical cellular processes. Over production of ROS (such H_2_O_2_) or inadequate antioxidant or scavenging enzyme activity could disrupt the cellular balance, which induces oxidative stress (Li et al., 2017; Yang et al., 2020b).

Both EOs (lemon and lemongrass) displayed abilities to induce scavenging as observed by the reduction of H_2_O_2_. The scavenging abilities of the essential oils may be ascribed to their chemical composition. Pino et al. (2018) reported the chemical composition of lemongrass whereby oxygenated monoterpenes and monoterpene hydrocarbons were the most represented class of compounds identified. According to Burits et al. (2001), monoterpene compounds identified in EOs may act as radical scavengers and EOs, which contain terpenes have greater antioxidative properties. As shown in this study in Figure 1, the content of H_2_O_2_ (one of the measured ROS) in EO treated *B. cinerea* was reduced in this study, although SOD was significantly higher in EOs treated *B. cinerea* mycelia compared to the control. In future studies, to fully understand the activity SOD, the level of total ROS should be determined. Overall, the results obtained suggests that the imbalance created by EOs, led to oxidative stress, and could be an important mechanism of antifungal activity.

### Effects of EOs on malondialdehyde in *B*. *cinerea* mycelia

Malondialdehyde is regarded as a end product of lipid peroxidation, and its excessive accumulation has been considered as a marker for oxidative stress (Mittler, 2002). The current study, showed that there were significant changes in the MDA content of essential oils treated *B*. *cinerea* when compared to the control (Figure 1B). Highest accumulation of MDA content was found in Lg-EO treated *B*. *cinerea*. The MDA content significantly increased in the Le-treatment (79%), Lg-treatment (278%), and in the combined Le + Lg (205%) in comparison to the untreated control at the end of day 7 (Figure 1B). Consistent with these current results, Khan et al. (2011) showed a 3-fold rise in MDA content in *Candida albicans* treated with eugenol, methyl eugenol and estragole, which are phenylpropanoid compounds found in EO.

Lipid peroxidation of cellular membrane is a complex process involving majorly, polyunsaturated fatty acids containing one or more methylene groups. These methylene groups are very sensitive to oxidizing agents resulting in the formation of peroxyl radicals, which can further cascade a sequence of reaction producing more free radicals and the accumulation of peroxidation by-products such as MDA (Avis et al., 2007). MDA can interact with DNA to form a propane adduct with 2’-deoxyguanosine. This complex has a significant impact on cell physiological activities this includes signaling, proliferation, differentiation, and death (Vasilaki and McMillan, 2011). Furthermore, it is reasonable to envisage the probable mode of action against *B. cinerea* involves oxidative stress as an antifungal activity of the EOs and their related compounds as shown in previous study. This activity could be associated with the activities of free radical scavenging agents, metal chelating and modification of cell signaling pathways (Khan et al., 2011). The increase in MDA content observed in our study in response to lemon and lemongrass EOs may thus be because of the EO constituents that bind to key enzymes on the membrane, causing membrane damage. This causes cell distortion and the loss of macromolecules from their interior which results in improper functioning of the cell membrane (Pramila et al., 2012). Therefore, EOs may induce damage to cellular protein, lipid, and nucleic acid content (Mani-López et al., 2021). Furthermore, each component of an EO contributes to the oil’s biological activity in its own way.

The major chemical compositions of EOs are terpenes, aldehydes, and phenols (Boubaker et al., 2016; Sharma and Kaur, 2022). Research have reported that EO’s with monoterpenes and sesquiterpenes exhibit antifungal activities against several fungi (Tian et al., 2015). Terpenes have been reported to induce membrane disruption which changes cell membrane permeability and fluidity and induces disturbances in membrane functions (Di Pasqua et al., 2007). Niu et al. (2022) also reported that the induction of MDA content of *A*. *niger* was associated with the inhibition of the ergosterol biosynthetic pathway, which led to plasma membrane damage and leakage of intracellular contents. In addition, ergosterol content was shown to be reduced in various fungal species treated with EOs (Mani-López et al., 2021). Ergosterol plays a role in regulating the activities of membrane-bound enzymes, membrane fluidity and permeability, as well as material transit by binding to phospholipids and stabilizing membrane structure (Krumpe et al., 2012). Tang et al. (2018) demonstrated that the geraniol and citral, which are major components of Lg EO effectively suppressed the growth of *A. flavus* and *A. ochraceus* by altering cell membrane permeability, causing electrolyte and cellular contents to leak, and interfered with key associated genes. These compounds are abundant in the Lg EO investigated in this study (Table 1).

### Effects of EOs on antioxidant enzyme activity in *B*. *cinerea*

In this study, total SOD activity of *B. cinerea* was assayed in response to the different EOs (Figure 1C). Given that SOD enzymatic activity catalyzes the conversion of O_2_^–^ to produce H_2_O_2_, it is expected that changes in H_2_O_2_ content could be attributed to alteration in SOD activity (Figure 1C). In response to the reduction in H_2_O_2_ content observed in Figure 1A, this study suggests the role of EOs in modulating *B. cinerea* SOD activity. Both Le- and Lg- EOs increased SOD activity in *B. cinerea* by 46% and 137% respectively, and highest increase in SOD activity (463%) was observed in the combination Le + Lg EO treatment when compared to the control non-treated *B. cinerea*.

Superoxide dismutase (SOD) is one of the key enzymes responsible for defense against oxidative stress (Yaseen et al., 2014; Valenzuela-Cota et al., 2019). Antioxidants operate as free radical scavengers and prevent free radical mediated processes. Khan et al. (2011) reported that the presence of EO components elevated the levels of SOD activity in *C*. *albicans*. Therefore, it is possible that the increase in SOD activity presented in this study may be attributed to the phenolic content that these EOs display. Based on the increased SOD activity in response to essential oils, it is reasonable to assume that H_2_O_2_ content would increase, since SOD can directly scavenge O_2_^–^ to produce H_2_O_2_. However, the current study showed that elevated levels of SOD activity was not associated with an increase in H_2_O_2_ content (Figure 1A).

Since EOs used in this study augmented SOD activity coupled with a reduction in H_2_O_2_ content, we investigated the effect of EOs on APX activity in *B. cinerea*. Given that APX catalyzes the conversion of H_2_O_2_ to water, we hypothesized that changes in APX activity would be highest in Lg treatment based on MDA content. This assumption was confirmed with APX activity in *B. cinerea* was highest in the Lg treatment with 206% increase followed the combination treatment (Le + Lg), with 73% in compared to the controls (Figure 1D). However, no significant difference in the APX enzymatic activity was observed in the Le treatment in comparison to the untreated control.

This work is the first to report APX activity in *B. cinerea* in response to Le and Lg EOs. Based on the results obtained from this work, we postulated that long progressive treatment of *B. cinerea* with Le and Lg EOs will reduce mycelia growth, and result in the development of differentially modulated biochemical responses. Furthermore, this study has shown that Lg EO is an efficient elicitor of *B. cinerea* response, as indicated by stress-induced oxidative damage (caused by enhanced MDA levels), which ultimately leads to membrane damage and cell death.

### 1D-SDS-PAGE oOf EOs induced *B. cinerea* mycelia protein

**FIGURE 2:**
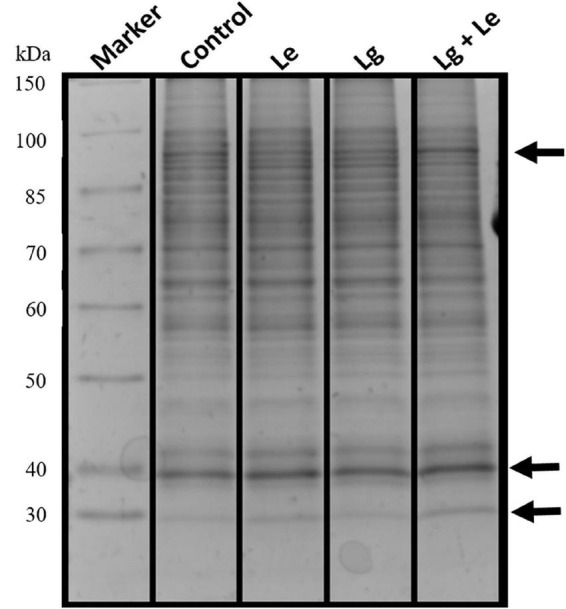
Protein extracts obtained from mycelia of *B. cinerea* exposed to different essential oils treatment fractionated on a 12% denaturing 1D SDS polyacrylamide gel showing separated proteins with no protein degradation.

Identification of protein via 1D gel electrophoresis has been demonstrated to show the presence of fractionated proteins based on molecular weight, the distance migrated and band intensity. Protein bands from all treatments covered a molecular weight range of 30 to 150 kDa (Figure 2). Separation and visualization of *B. cinerea* demonstrated that the proteins are of high quality with no visible streaking or protein degradation. Bands of fragmented or fractionated proteins between the two EOs and their combination showed some similarity to the control based on their intensity and banding patterns. However, changes were observed in the protein profiles between treatments (indicated by arrows), with protein bands at ≈30, ≈40 and ≈90 kDa were shown to have higher band intensity suggesting possible over-expression or up regulation of proteins within these bands in the treated *B. cinerea*. Variation in protein intensity in this study could suggest that the protein expression in the non-treated control was down-regulated (Nsumpi et al., 2020).

### Identification of induced proteins in *B. cinerea* using LC-MS

The blank injection before the analysis showed no major contaminants judging by the total ion chromatogram (TIC; Supplementary Figure 1A). While the system suitability sample (a four-protein mixture) indicated successful digestion and demonstrated that peptides were both retained and eluted as expected judging by the TIC (Supplementary Figure 1B). As a critical component of bio-analysis was performed based on the data generated for the blank and the system suitability sample. All samples were analyzed and captured as individual TICs (Supplementary Figure 2). While the TICs indicated successful digestion on the system suitability sample (a four-protein mixture) as analyzed on the mass spectrometer, the combination treatment seemed to have less material or was more difficult to digest judging by the number of peaks and signal intensity (Supplementary Figure 2D).

**FIGURE 3:**
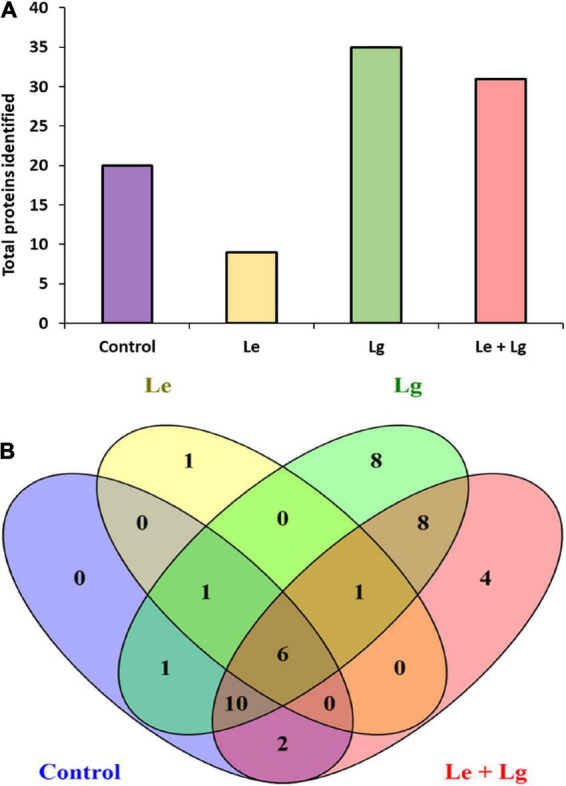
Detection of proteins in the mycelia of *B. cinerea* in response to lemon and lemongrass EOs exposure. **(A)** Total number of proteins identified in the different treatments, and **(B)** Venn diagram illustrating the common and unique proteins identified in all treatments and control.

Data base interrogation of the total ion chromatography for all samples against the Uniprot database for *Botrytis* was performed, and 110 proteins with 7 clusters was identified with an FDR of 0.95% (Figure 3A). To ensure correct identification of proteins, a selection threshold of 95% was set for the identified proteins. Furthermore, the protein exclusive unique peptide count should be ≥ 2 for the protein to be considered as a positive identification; and the identification probability should be ≥ 95%, and the percentage sequence coverage should > 0. Based on these selection and identification parameters, 42 proteins were confidently identified in this study, with 20 proteins identified in the control, 9 in Le-treatment, 35 in Lg-treatment, and 31 in combined treatment (Le + Lg)) as shown in Figure 3A and Table 2.

**TABLE 2:** List of proteins identified in lemon and lemongrass essential oil treated *B. cinerea* mycelia this study.

Identified proteins	Accession number	Entry name	Protein present			
			Control	Le	Lg	Le + Lg
**Binding proteins**						
Actin	O13419	ACT_BOTFU	100	100	100	100
ATPase get3	A6S7T2	GET3_BOTFB	100	100	100	100
Cyanate hydratase	A6S6V7	CYNS_BOTFB	100	100	100	100
Mitogen-activated protein kinase hog1	A1IVT7	HOG1_BOTFB	99	-	100	100
Adenylate kinase	A6RPU0	KAD2_BOTFB	100	55	100	100
Clustered mitochondria protein homolog	A7ENU3	CLU_SCLS1	100	-	95	100
Vacuolar membrane protease	A6S8A1	PFF1_BOTFB	100	-	100	100
Probable Xaa-Pro aminopeptidase pepP	A6SDE9	AMPP3_BOTFB	-	100	-	-
Histone H2A	O74268	H2A_BOTFB	-	100	100	100
Protein sey1	A6S544	SEY1_BOTFB	80	-	100	-
**Catalytic proteins**						
Probable beta-galactosidase B	A6RPN7	BGALB_BOTFB	100	100	100	-
Probable dipeptidyl-aminopeptidase B	A6SL49	DAPB_BOTFB	95	-	100	100
NADH-cytochrome b5 reductase 2	A6SI59	MCR1_BOTFB	100	-	100	100
Arginine biosynthesis bifunctional protein ArgJ 1, mitochondrial	A6S146	ARGJ1_BOTFB	-	-	100	100
Endo-1,4-beta-xylanase 11A	Q2LMP0	XY11A_BOTFU	100	-	-	100
**RNA helicase proteins**						
ATP-dependent RNA helicase dbp5	A6SBT4	DBP5_BOTFB	95	-	95	100
ATP-dependent RNA helicase dbp2	A6SFW7	DBP2_BOTFB	100	-	100	100
ATP-dependant RNA helicase eIF4A	A6RJ45	IF4A_BOTFB	100	100	100	100
ATP-dependant RNA helicase ded1	A6SEH9	DED1_BOTFB	99	-	100	100
ATP-dependant RNA helicase sub2	A7EIX7	SUB2_SCLS1	-	-	100	95
ATP-dependant RNA helicase dhh1	A6RY31	DHH1_BOTFB	-	-	100	100
**Structure molecule**						
Tubulin beta chain	P53373	TBB_BOTFU	100	100	100	100
40S ribosomal protein S1	A6RZS5	RS3A_BOTFB	100	37	18	100
**Translation initiation factor proteins**						
Eukaryotic translation initiation factor 3 subunit I	A6RUL1	EIF3I_BOTFB	-	-	100	-
Eukaryotic translation initiation factor 3 subunit D	A6SJW6	EIF3D_BOTFB	-	-	100	-
Eukaryotic translation initiation factor 3 subunit B	A6SFQ6	EIF3B_BOTFB	-	-	100	100
Eukaryotic translation initiation factor 3 subunit E	A6SM77	EIF3E_BOTFB	-	-	100	-
Eukaryotic translation initiation factor 3 subunit L	A6S1A3	EIF3L_BOTFB	-	-	100	44
**G protein activity**						
Ras-like protein	P87018	RAS_BOTFU	100	100	100	100
**Transmembrane transporter activity**						
Mitochondrial glycine transporter	A7F9Y3	S2538_SCLS1	100	-	100	-
**Unknown**						
MICOS complex subunit mic60	A7F6C1	MIC60_SCLS1	100	-	100	100
Autophagy-related protein 3	M7UQV4	ATG3_BOTF1	-	-	100	95
Nucleolar protein 58	A6RMY5	NOP58_BOTFB	-	-	100	-
protein rot1	A6S3W1	ROT1_BOTFB	-	-	100	-
Secreted RxLR effector protein 144	P0CV60	RL144_PLAVT	-	-	-	100
Nascent polypeptide-associated complex subunit alpha	A6SB28	NACA_BOTFB	-	-	-	100
Mitochondrial division protein 1	A7ETB3	MDV1_SCLS1	-	-	-	100
**Microtubule motor activity**						
Kinesin heavy chain	Q86ZC1	KINH_BOTFU	-	-	100	100
**Antioxidant activity**						
Superoxide dismutase [Cu-Zn]	Q70Q35	SODC_BOTFU	76	-	100	100
Catalase A	P55304	CATA_BOTFU	-	-	95	-
**Protein macromolecule adaptor activity**						
Actin cytoskeleton-regulatory complex protein sla1	A7E8B6	SLA1_SCLS1	-	-	100	100
protein sds23	A7F3V4	SDS23_SCLS1	58	-	-	100

Values marked in red fonts were below the 95% threshold.

Protein accessions identified in this study (Table 2) were analyzed with the FunRich Multi analysis software (version 3.1.3), to distinguish proteins there were common and/or unique in each of the treatments (Figure 3B). Results showed that 13 of 42 proteins identified in this study were unique, with 0 – control; 1 – Le; 8 – Lg; and 4 – (Le + Lg) EOs (Figure 3B). On the other hand, 6 proteins (ACT BOTFU, GET3 BOTFB, CYNS BOTFU, RAS BOTFU, IF4A BOTFB and TBB BOTFU) were conserved in all treatments. Seven proteins were shared between untreated control and Le-treatments, while untreated control and Lg-treatment shared 18 proteins. For the comparison of control and combination treatment (Le + Lg) EO, 18 proteins were shared between untreated control and combined (Le + Lg) EOs treated *B. cinerea* (Figure 3B).

### Subcellular localization of responsive proteins of *B. cinerea*

**FIGURE 4:**
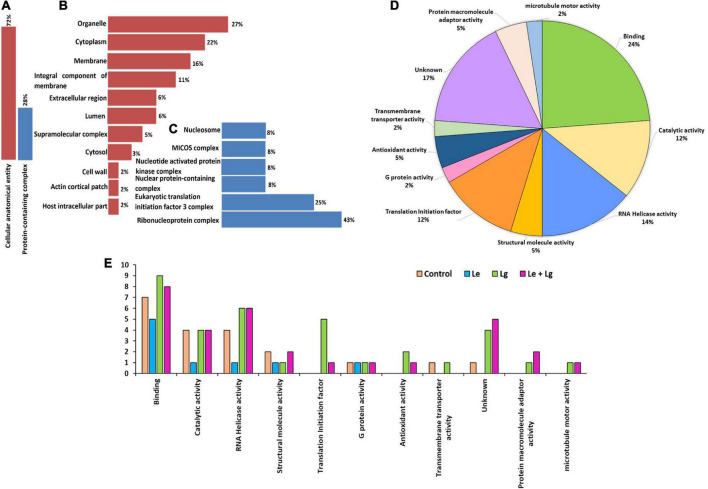
Cellular compartment analysis of the total number of proteins identified: **(A)** Percentage distribution of cellular compartments in the cellular anatomical entity, **(B)** shows a further analysis of 72%, and **(C)** sub-cellular representation in the protein-containing complex, **(D)** functional and molecular classification of essential oil responsive proteins identified in *B. cinerea* and **(E)** gene ontology (GO) classification annotation of DEPs for treated and untreated *B. cinerea* and the number of identified proteins for various treatments on *y*-axis. The functions were categorized according to gene ontology annotations from the UniProt database (https://www.uniprot.org/).

Proteomics plays a major role in the nature and uses of essential oils on fungi. The subcellular localization (Figure 4) helped in further determining key functional characteristic of protein-protein interaction. Our study showed that 72% of proteins identified were localized within the cellular anatomical entity, and 28% of proteins in the protein-containing complex (Figure 4A). Subcellular localization results showed that major proteins were widely distributed within organelles (27%) and cytoplasm (22%). Partitioning of the proteome between organelle and cytoplasm affects nearly every aspect of eukaryotic biology (Emanuelsson, 2002), and performs a wide range of metabolic functions, which play vital role in cellular integrity (Zybailov et al., 2008; Walker et al., 2018). This could be a major reason why most of the identified proteins were predominantly located in organelles and cytoplasm.

In response to chemical stress and defense, cellular integrity is essential for fungal adaptation and survival. The response to chemical stress causes fungal disruption which induces the efflux of cytoplasmic constituents (Liu et al., 2017). Notably, the subcellular location of approximately 16% of the identified proteins was in the membrane and some were identified in the integral component of the membrane (11%). The rest of the proteins were found in the extracellular region (6%), lumen (6%), supramolecular complex (5%), cytosol (3%), cell wall (2%), actin cortical patch (2%) and host intracellular parts (2%) (Figure 4B). Proteins localized in the protein-containing complex (Figure 4A) were further sub-localized in the ribonucleoprotein complex (43%), initiation factor 3 complex (25%) and the remaining proteins each had 8% belonging to its subclass or group (Figure 4C).

### Protein-protein interaction and molecular functional specificity

The subcellular localization (Figure 4) helped in further determining key functional characteristics of protein-protein interaction (Figures 4D–E). Since most proteins interact with other proteins for proper function, it is important that they should be investigated in concert (within the context of their interactions) to fully understand their biological functions. Similarly, to successfully assign functions to unknown proteins the complete interaction should be understood (Xenarios and Eisenberg, 2001; Kim et al., 2002). GO enrichment analysis was used to understand protein functions. The identified proteins were analyzed by GO annotation via the Uniprot database (http://www.uniprot.org) and categorized based on functionality into 11 different groups (Figure 4D). Majority of the enriched proteins were related with binding (24%) followed by proteins with unknown functions (17%), RNA helicase activity (14%), catalytic activity (12%) and translation initiation factor (12%). A few of the proteins identified were also involved in antioxidant activity (5%), structural molecules (5%), protein macromolecule adaptor activity (5%), G protein activity (2%), transmembrane transporter activity (2%) and microtubule motor activity (2%).

The current proteomics results also showed changes in the total proteins identified in the different EO-treatments for each category (Figure 4E). Treatment with Lg and its binary mixture (Le + Lg) increased the number of proteins involved in binding mechanisms, catalytic activity, and proteins with no known function. However, proteins involved in RNA Helicase activity were affected by treatment with Lg and the binary mixture (Le + Lg) as observed the numbers proteins involved in RNA Helicase activity were higher in Lg and (Le + Lg) EOs treated in *B. cinerea* mycelia compared to the Le EO treated and control samples. Furthermore, some proteins involved in translation initiation, antioxidant activity, protein macromolecule adaptor activity and microtubule motor activity were only identified in the Lg and combination treatment.

### Comparative analysis of proteins under various treatment of EOs

#### Binding proteins

The comparative proteomics analyses of binding proteins revealed that one Probable Xaa-Pro (Prolidase, A6SDE9) was specifically found in Le treatment (Supplementary Table 2). This indicates that prolidase was activated in response to Le treatment (Supplementary Table 2). According to Wilk et al. (2021), prolidase is postulated as being involved in nutrition acquisition, and antitoxin defense in prokaryotes. Prolidase has been shown to contribute to osmoregulation by releasing free proline (Zaprasis et al., 2013), and prolidase variations have been linked to antibiotic resistance (Wilk et al., 2021). Prolidases are believed to have a broad antitoxin protective role due to their capacity to hydrolyse organophosphorus chemicals (Štěpánková et al., 2013). It is noteworthy that the activities of prolidase in *B. cinerea* is not entirely understood, and the mechanism of inhibiting *B. cinerea* growth by Le is still unclear. This led us to suggest that Le might be inhibiting *B. cinerea* growth through alteration of nutrient uptake and the fungi might be activating prolidase to acquire nutrient for its survival under stressed conditions.

Interestingly, our comparative proteomics analyses of binding proteins under Lg treatment revealed one unique protein, which was Sey1 protein. Sey1/atlastin, is a dynamin-like GTPase protein that is considered essential for homotypic fusion of ER membranes, however, its activities in pathogenic fungi remain unclear. Chong et al. (2020) previously established that FgSey1 in *F*. *graminearum*, and an homologue of Sey1/atlastin, is necessary for vegetative development, DON synthesis and pathogenicity. Their findings shed light on the crucial functions of FgSey1 in fungal pathogenicity, implying that FgSey1 could be considered a potential marker for effective *F*. *graminearum* disease management.

#### RNA helicase

RNA helicase activity plays an important role during cell growth and involves an ATP-driven unwinding of an RNA duplex. These enzymes, forms part of the *DEAD* box protein family defined by the amino acid sequence aspartic acid, glutamic acid, alanine and aspartic acid (*DEAD*). The ATP-dependant RNA helicase proteins ded1, dbp2 and dbp5 were found to be unique to the control (Supplementary Table 2). Studies have shown that both ded1 and dbp2 participate in translation initiation (Chuang et al., 1997; de la Cruz et al., 1997), in processing rRNA as well as nonsense-mediated mRNA decay (Bond et al., 2001). Studies done by Grallert et al. (2000) has also shown that ded1 expression is necessary for the translation of several important cell cycle proteins. Liu et al. (2002) confirmed that inactivation of ded1 causes cell cycle arrest.

Therefore, in this study, treatment with essential oils (Le + Lg) and Lg alone, up regulated these proteins suggesting that the *B. cinerea* is actively trying to grow after the treatments possibly induced cell cycle arrest and growth inhibition. Furthermore, *eIF4A* was unique to the Le treatment. Eukaryotic initiation factors (*eIFs*) are proteins that aid the start of eukaryotic translation. They help to construct a functioning ribosome around the start codon, and support regulatory mechanisms needed to initiate translation and protein synthesis (Chu et al., 2016). *Sub2* and *dhh1* were also activated in response to Lg- and the combined EO treatments and were not found in Le EO treated and control samples. These co-regulated proteins indicate that the initiation of translation and signaling pathways evoked by these structurally distinct proteins are integrated, and this could fulfil related tasks in *B. cinerea*, which is of potential interest in understanding the complex signaling role played by these EOs.

#### Antioxidant and other functional proteins

Based on the identified functional proteins superoxide dismutase (Q70Q35, SODC_BOTFU) and catalase A (P55304, CATA_BOTFU) linked to antioxidant activity in this study were found to be upregulated. These proteins are known for their role in the defense against ROS species and SOD was identified and quantified in this study. Superoxide dismutase (Q70Q35) protein was detected in the control samples and in the Lg and the combined (Le + Lg) EOs treated *B. cinerea* (Table 2). Notably, the detection of superoxide dismutase (Q70Q35) in the control sample was below the set threshold, hence, it was considered absent. In contrast, catalase A (P55304) was only found in the Lg EO treated *B. cinerea* samples. Reactive oxygen species (ROS) have the potential to bring about direct injury to biological elements such as membrane lipids, the photosystem II complex (Smirnoff, 1993; Chen and Murata, 2002; Mittler, 2002), proteins and DNA (Xiong and Zhu, 2002). Intracellular levels of ROS are maintained by several mechanisms, which collectively constitute the antioxidant response. ROS scavenging enzymes such as superoxide dismutase (SOD) (dismutase O_2_^∙–^ into H_2_O_2_), glutathione peroxidase (GPX) (uses glutathione to breakdown H_2_O_2_ to H_2_O) and catalase (CAT) (decomposes H_2_O_2_ to H_2_O) are ubiquitous effectors of the enzyme-mediated antioxidant response (Xiong and Zhu, 2002).

Other proteins that were found in the *B. cinerea mycelia* such as protein sds23 (A7F3V4), have been shown to be uniquely found in the combined Le + Lg EO treatment, and was above set threshold. Similarly, actin cytoskeleton-regulatory complex protein sla1 (A7E8B6) was only found in Lg and combined Le + Lg EO treatment (**Tables 2**, S2). Although, this protein’s particular function is unknown, it is assumed to be essential for normal DNA replication and mitosis (Ishii et al., 1996; Jang et al., 1997). It has also been found to boost sexual development as well as to have a role in the stress response to dietary limitation (Goldar et al., 2005; Yakura et al., 2006). Phosphorylation appears to be key in controlling protein Sds23 function; the protein is largely dephosphorylated in rapidly developing cells, but primarily phosphorylated in stationary phase. Other unique proteins were identified, this includes proteins 1 (A7ETB3, mitochondrial division), protein 3 (M7UQV4, autophagy-related), protein rot1 (A6S3W1) and nuclear protein 58 (A6RMY5). However, as result of limited information about *B. cinerea* in the public domain and in various databases, these proteins were classified with unknown molecular functions. Further research is needed that can better characterize these unknown proteins and to understand their biological functions.

## Conclusion

This study highlighted the chemical composition of lemon and lemongrass essential oils (EOs) and provided novel insight into their induced enzymatic and proteomic response in treated *Botrytis cinerea*. Monoterpene hydrocarbons (97%) and oxygenated monoterpenes (94%) were the predominant constituent of lemon and lemongrass EOs, respectively. Based on the enzymatic bioassays the presence and accumulation of superoxide dismutase (SOD), ascorbate peroxidase (APX), and malondialdehyde (MDA) were confirmed in the EO-treated *B. cinerea* compared to non-treated control. High concentrations of APX under Lg and Le + Lg treatments could suggest that ROS was induced by the EOs, and this caused direct damage to biological components such as membrane lipids releasing the cellular entity and protein-complexes (based on the proteomic results). Lemongrass and Le + Lg EOs treatments were identified as the most consequential elicitors of *B. cinerea* responses, based on the induced oxidative stress damage (caused by enhanced MDA levels). The detection of proteins linked to RNA helicase, transmembrane transporter, and antioxidant activities in EO-treated *B. cinerea* above the set threshold further validates the bioassay results. Proteins such as catalase (P55304) and superoxide dismutase [Cu-Zn] (Q70Q35) associated antioxidant activities upregulated in EO-treated *B. cinerea.* These proteins are responsible for regulating ROS, which protects cellular components from oxidative stress) and could have played crucial role in the cell inactivation process. Our results provide an enzymatic and proteomic reference for future research into the role of EOs as effective anti-*B. cinerea* agent.

## Data availability statement

The proteomics data presented in the study are deposited to the ProteomeXchange Consortium via the PRIDE partner repository (doi: 10.6019/PXD038894) with the dataset identifier PXD038894.

## Author contributions

IK was responsible for conducting the experiments and drafted the first manuscript. ZB, AK, and GM supervised the data collection and analysis. GM contributed to the data analysis and the optimization of protein extractions. PM, ZB, and OC contributed to the development of the experimental design, data analysis, and proofreading. IK, PM, AK and OC contributed to the interpreted results. PM, AK, and OC designed the study. AK and OC were the lead investigators, supervised the students, and final proofreading. All authors contributed to the article and approved the submitted version.

## References

[B1] Artés-Hernández F. Aguayo E. Artés F. (2004). Alternative atmosphere treatments for keeping quality of ‘Autumn Seedless’ table grapes during long-term cold storage. *Postharv. Biol. Technol.* 31 59–67. 10.1016/S0925-5214(03)00116-9

[B2] Artés-Hernández F. Tomás-Barberán F. A. Artés F. (2006). Modified atmosphere packaging preserves quality of SO2-free ‘Superior Seedless’ table grapes. *Postharv. Biol. Technol.* 39 146–156. 10.1016/j.postharvbio.2005.10.006

[B3] Asada K. (1992). Ascorbate peroxidase - a hydrogen peroxide scavenging enzyme in plants. *J. Plant Physiol.* 85 235–241.

[B4] Avis T. J. Michaud M. Tweddell R. J. (2007). Role of lipid composition and lipid peroxidation in the sensitivity of fungal plant pathogens to aluminum chloride and sodium metabisulfite. *Appl. Environ. Microbiol.* 73 2820–2824. 10.1128/AEM.02849-06 17337539 PMC1892857

[B5] Belay Z. A. Caleb O. J. (2022). Role of integrated omics in unravelling fruit stress and defence responses during postharvest: A review. *Food Chem. Mol. Sci.* 5:100118. 10.1016/j.fochms.2022.100118 PMC9278069 35845150

[B6] Beyer W. F. Fridovich Y. (1987). Assaying for superoxide dismutase activity: Some large consequences of minor changes in conditions. *Anal. Biochem.* 161 559–566. 10.1016/0003-2697(87)90489-1 3034103

[B7] Bianco L. Perrotta G. (2015). Methodologies and perspectives of proteomics applied to filamentous fungi: From sample preparation to secretome Analysis. *Int. J. Mol. Sci.* 16 5803–5829. 10.3390/ijms16035803 25775160 PMC4394507

[B8] Bond A. T. Mangus D. A. He F. Jacobson A. (2001). Absence of Dbp2p alters both nonsense-mediated mRNA decay and rRNA processing. *Mol. Cell. Biol.* 21 7366–7379. 10.1128/MCB.21.21.7366-7379.2001 11585918 PMC99910

[B9] Boubaker H. Karim H. Elhamdaoui A. Msanda F. Leach D. Abbad A. (2016). Chemical characterization and antifungal activities of four Thymus species essential oils against postharvest fungal pathogens of citrus. *Indus. Crops Prod.* 86 95–101. 10.4315/0362-028x-64.7.1025 11456187

[B10] Brink J. C. Calitz F. J. Fourie P. H. (2016). Spray deposition and control of *Botrytis cinerea* on grape leaves and bunches: Part 1 (Table Grapes). *South African J. Oenol. Viticul.* 37 93–103.

[B11] Brochot A. Guilbot A. Haddioui L. Roques C. (2017). Antibacterial, antifungal, and antiviral effects of three essential oil blends. *Microbiologyopen* 6:e00459. 10.1002/mbo3.459 28296357 PMC5552930

[B12] Burits M. Asres K. Bucar F. (2001). The antioxidant activity of the essential oils of Artemisia afra, *Artemisia abyssinica* and *Juniperus procera*. *Phytother. Res.* 15 103–108. 11268106 10.1002/ptr.691

[B13] Chen T. H. Murata N. (2002). Enhancement of tolerance of abiotic stress by metabolic engineering of betaines and other compatible solutes. *Curr. Opin. Plant Biol.* 5 250–257. 10.1016/s1369-5266(02)00255-8 11960744

[B14] Cheung N. Tian L. Liu X. Li X. (2020). The destructive fungal pathogen *Botrytis cinerea*-insights from genes studied with mutant analysis. *Pathogens* 7:923. 10.3390/pathogens9110923 33171745 PMC7695001

[B15] Chong X. Wang C. Wang Y. Wang Y. Zhang L. Liang Y. (2020). The dynamin-like GTPase FgSey1 plays a critical role in fungal development and virulence in *Fusarium graminearum*. *Appl. Environ. Microbiol.* 86 e2720–e2719. 10.1128/AEM.02720-19 32220839 PMC7237792

[B16] Chu J. Galicia-Vázquez G. Cencic R. Mills J. R. Katigbak A. Porco J. A. Jr. (2016). CRISPR-mediated drug-target validation reveals selective pharmacological inhibition of the RNA helicase, eIF4A. *Cell Rep.* 15 2340–2347. 10.1016/j.celrep.2016.05.005 27239032 PMC5315212

[B17] Chuang R.-Y. Weaver P. L. Liu Z. Chang T.-H. (1997). Requirement of the DEAD-box protein Ded1p for messenger RNA translation. *Science* 275 1468–1471. 10.1126/science.275.5305.1468 9045610

[B18] de la Cruz J. Iost I. Kressler D. Linder P. (1997). The p20 and Ded1 proteins have antagonistic roles in eIF4E-dependent translation in Saccharomyces cerevisiae. *Proc. Natl. Acad. Sci. U.S.A.* 94 5201–5206. 10.1073/pnas.94.10.5201 9144215 PMC24656

[B19] de Oliveira Filho J. G. da Cruz Silva G. de Aguiar A. C. Cipriano L. de Azeredo H. M. C. Junior S. B. (2021). Chemical composition and antifungal activity of essential oils and their combinations against Botrytis cinerea in strawberries. *Food Meas.* 15 1815–1825.

[B20] Di Pasqua R. Betts G. Hoskins N. Edwards M. Ercolini E. Mauriello G. (2007). Membrane toxicity of antimicrobial compounds from essential oils. *J. Agric. Food Chem.* 55 4863–4870. 17497876 10.1021/jf0636465

[B21] Dwyer D. J. Kohanski M. A. Collins J. J. (2009). Role of reactive oxygen species in antibiotic action and resistance”. *Curr. Opin. Microbiol.* 2 482–489. 10.1016/j.mib.2009.06.018 PMC2761529 19647477

[B22] Egbichi I. Keyster M. Jacobs A. Klein A. Ludidi N. (2013). Modulation of antioxidant enzyme activities and metabolites ratios by nitric oxide in short-term salt stressed soybean root nodules. *South African J. Bot.* 88 326–333.

[B23] Emanuelsson O. (2002). Predicting protein subcellular localization from amino acid sequence information. *Brief. Bioinform.* 3 361–376. 10.1093/bib/3.4.361 12511065

[B24] Fernández-Acero F. J. Colby T. Harzen A. Cantoral J. M. Schmidt J. (2009). Proteomic analysis of the phytopathogenic fungus Botrytis cinerea during cellulose degradation. *Proteomics* 9 2892–2902. 10.1002/pmic.200800540 19415670

[B25] Fernández-Acero F. J. Colby T. Harzen A. Carbu M. Wiencke U. Cantoral J. M. (2010). 2-DE proteomic approach to the Botrytis cinerea secretome induced with different carbon sources and plant-based elicitors. *Proteomics* 10 2270–2280. 10.1002/pmic.200900408 20376862

[B26] Fernández-Acero F. J. Jorge I. Calvo E. Vallejo I. Carbu M. Camafeita E. (2006). Two-dimensional electrophoresis protein profile of the phytopathogenic fungus *Botrytis cinerea*. *Proteomics* 6 (Suppl. 1) 88–96. 10.1002/pmic.200500436 16544282

[B27] Gakuubi M. M. Maina A. W. Wagacha J. M. (2017). Antifungal activity of essential oil of Eucalyptus camaldulensis Dehnh against selected Fusarium spp. *Int. J. Microbiol.* 2017:8761610. 10.1155/2017/8761610 28127308 PMC5239988

[B28] Goldar M. M. Nishie T. Ishikura Y. Fukuda T. Takegawa K. Kawamukai M. (2005). Functional conservation between fission yeast moc1/sds23 and its two orthologs, budding yeast SDS23 and SDS24, and phenotypic differences in their disruptants. *Bio. Biotechnol. Biochem.* 69 1422–1426. 10.1271/bbb.69.1422 16041152

[B29] Grallert B. Kearsey S. E. Lenhard M. Carlson C. R. Nurse P. Boye E. (2000). A fission yeast general translation factor reveals links between protein synthesis and cell cycle controls. *J. Cell Sci.* 113 1447–1458. 10.1242/jcs.113.8.1447 10725227

[B30] Grinyer J. McKay M. Nevalainen H. Herbert B. R. (2004). Fungal proteomics: Initial mapping of biological control strain *Trichoderma harzianum*. *Curr. Gen.* 45 163–169. 10.1007/s00294-003-0474-4 14685766

[B31] Ishii H. Jirousek M. R. Koya D. Takagi C. Xia P. Clermont A. (1996). Amelioration of vascular dysfunctions in diabetic rats by an oral PKC β inhibitor. *Sci.* 272 728–731. 10.1126/science.272.5262.728 8614835

[B32] Jang J. C. León P. Zhou L. Sheen J. (1997). Hexokinase as a sugar sensor in higher plants. *Plant Cell* 9 5–19. 9014361 10.1105/tpc.9.1.5 PMC156897

[B33] Kalagatur N. K. Ghosh O. S. N. Sundararaj N. Mudili V. (2018). Antifungal activity of chitosan nanoparticles encapsulated with *Cymbopogon martinii* essential oil on plant pathogenic fungi Fusarium graminearum. *Front. Pharmacol.* 9:610. 10.3389/fphar.2018.00610 29928233 PMC5997812

[B34] Kgang I. E. Mathabe P. M. K. Klein A. Kalombo L. Belay Z. A. Caleb O. J. (2022). Effects of lemon (*Citrus limon* L.), lemongrass (*Cymbopogon citratus*) and peppermint (*Mentha piperita* L.) essential oils on mycelial growth and spore germination of *Botrytis cinerea* and *Penicillium expansum* – In vitro study. *J. Sci. Food Agric. Rep.* 2 405–414.

[B35] Khan A. Ahmad A. Akhtar F. Yousuf S. Xess I. Khan L. A. (2011). Induction of oxidative stress as a possible mechanism of the antifungal action of three phenylpropanoids. *FEMS Yeast Res.* 11 114–122. 10.1111/j.1567-1364.2010.00697.x 21114624

[B36] Kim J. Krapivsky P. L. Kahng B. Redner S. (2002). Infinite-order percolation and giant fluctuations in a protein interaction network. *Phys. Rev. E* 66:055101. 10.1103/PhysRevE.66.055101 12513542

[B37] Krumpe K. Frumkin I. Herzig Y. Rimon N. Özbalci C. Brügger B. (2012). Ergosterol content specifies targeting of tail-anchored proteins to mitochondrial outer membranes. *Mol. Biol. Cell* 23 3927–3935. 10.1091/mbc.E11-12-0994 22918956 PMC3469509

[B38] Li Y. Shao X. Xu J. Wei Y. Xu F. Wang H. (2017). Tea tree oil exhibits antifungal activity against Botrytis cinerea by affecting mitochondria. *Food Chem.* 234 62–67. 10.1016/j.foodchem.2017.04.172 28551268

[B39] Liu H. Y. Nefsky B. S. Walworth N. C. (2002). The Ded1 DEAD box helicase interacts with Chk1 and Cdc2. *J. Biol. Chem.* 277 2637–2643. 10.1074/jbc.M109016200 11711540

[B40] Liu P. Luo L. Guo J. Liu H. Wang B. Deng B. (2010). Farnesol induces apoptosis and oxidative stress in the fungal pathogen *Penicillium expansum*”. *Mycologia* 102 311–318. 10.3852/09-176 20361499

[B41] Liu X. Ma Z. Zhang J. Yang L. (2017). Antifungal compounds against Candida infections from traditional Chinese medicine”. *BioMed. Res. Int.* 2017:4614183. 10.1155/2017/4614183 29445739 PMC5763084

[B42] Mani-López E. Cortés-Zavaleta O. López-Malo A. (2021). A review of the methods used to determine the target site or the mechanism of action of essential oils and their components against fungi. *SN Appl. Sci.* 3:44. 10.1007/s42452-020-04102-1

[B43] Mathabe P. M. K. Belay Z. A. Ndlovu T. Caleb O. J. (2020). Progress in proteomic profiling of horticultural commodities during postharvest handling and storage: A Review. *Sci. Hortic.* 261:108996. 10.1016/j.scienta.2019.108996

[B44] Mittler R. (2002). Oxidative stress, antioxidants, and stress tolerance. *Trends Plant Sci.* 7 405–410. 12234732 10.1016/s1360-1385(02)02312-9

[B45] Moutassem D. Belabid L. Bellik Y. Ziouche F. B. (2019). Efficacy of essential oils of various aromatic plants in the biocontrol of Fusarium wilt and inducing systemic resistance in chickpea seedlings. *Plant Prot. Sci.* 55 202–217.

[B46] Munhuweyi K. Caleb O. J. Lennox C. L. van Reenen A. J. Opara U. L. (2017). In vitro and in vivo antifungal activity of chitosan-essential oils against pomegranate fruit pathogens. *Postharv. Biol. Technol.* 129 9–22.

[B47] Niu A. Wu H. Ma F. Tan S. Wang G. Qiu W. (2022). The antifungal activity of cinnamaldehyde in vapor phase against Aspergillus niger isolated from spoiled paddy. *Food Sci. Technol.* 159:113181. 10.1016/j.lwt.2022.113181

[B48] Nsumpi A. N. Belay Z. A. Caleb O. J. (2020). Good intentions, bad outcomes: Impact of mixed-fruit loading on banana fruit protein expression, physiological responses and quality. *Food Packag. Shelf Life* 26:100594. 10.1016/j.fpsl.2020.100594

[B49] Oliveros J. C. (2007-2015). *Venny. “An interactive tool for comparing lists with Venn’s diagrams.* Available online at: https://bioinfogp.cnb.csic.es/tools/venny/index.html (accessed July 24, 2022).

[B50] Ong S. E. Pandey A. (2001). An evaluation of the use of two-dimensional gel electrophoresis in proteomics. *Biomol. Eng.* 18 195–205. 11911086 10.1016/s1389-0344(01)00095-8

[B51] OuYang Q. Liu Y. Oketch O. R. Zhang M. Shao X. Tao N. (2021). Citronellal exerts its antifungal activity by targeting ergosterol biosynthesis in *Penicillium digitatum*. *J. Fungi.* 7:432. 10.3390/jof7060432 34072578 PMC8229684

[B52] Perez-Riverol Y. Bai J. Bandla C. Hewapathirana S. García-Seisdedos D. Kamatchinathan S. (2022). The PRIDE database resources in 2022: A Hub for mass spectrometry-based proteomics evidences. *Nucleic Acids Res.* 50 D543–D552. 10.1093/nar/gkab1038 34723319 PMC8728295

[B53] Pino J. A. Fon-Fay F. M. Pérez J. C. Falco A. S. Rodríguez J. L. Hernández I. (2018). Chemical composition and biological activities of essential oil from lemongrass (*Cympopogon citratus* [D.C.] Stapf.) leaves grown in Amazonian Ecuador. *Rev. CENIC* 49. Available online at: https://www.redalyc.org/articulo.oa?id=181661081008

[B54] Powers C. N. Osier J. L. McFeeters R. L. Brazell C. B. Olsen E. L. Moriarity D. M. (2018). Antifungal and cytotoxic activities of sixty commercially-available essential oils. *Molecules* 23:1549. 10.3390/molecules23071549 29954086 PMC6100473

[B55] Pramila D. M. Xavier R. Marimuthu K. Kathiresan S. Khoo M. L. Senthilkumar M. (2012). Phytochemical analysis and antimicrobial potential of methanolic leaf extract of peppermint (*Mentha piperita*: Lamiaceae). *J. Medic. Plants Res.* 6 331–335.

[B56] Qin G. Liu J. Cao B. Li B. Tian S. (2011). Hydrogen peroxide acts on sensitive mitochondrial proteins to induce death of a fungal pathogen revealed by proteomic analysis. *PLoS One* 6:e21945. 10.1371/journal.pone.0021945 21755012 PMC3130790

[B57] Röhrig H. Schmidt J. Colby T. Brautigam A. Hufnagel P. Bartels D. (2006). Desiccation of the resurrection plant Craterostigma plantagineum induces dynamic changes in protein phosphorylation. *Plant Cell Environ.* 29 1606–1619. 10.1111/j.1365-3040.2006.01537.x 16898021

[B58] Shah B. B. Mehta A. A. (2018). In vitro evaluation of antioxidant activity of d–limonene. *Asian J. Pharm. Pharmacol.* 4 883–887.

[B59] Sharma A. D. Kaur I. (2022). Essential oil from Cymbopogon citratus exhibits “anti-aspergillosis” potential: In-silico molecular docking and in vitro studies. *Bull. National Res. Cen.* 46 1–15. 10.1186/s42269-022-00711-5 35125860 PMC8800409

[B60] Smirnoff N. (1993). The role of active oxygen in the response of plants to water deficit and desiccation. *New Phytol.* 125 27–58. 33874604 10.1111/j.1469-8137.1993.tb03863.x

[B61] Štěpánková A. Dušková J. Skálová T. Hašek J. Koval T. Østergaard L. H. (2013). Organophosphorus acid anhydrolase from Alteromonas macleodii: Structural study and functional relationship to prolidases”. *Acta Crystal. Sec. F Struc. Biol. Crystal. Commun.* 69 346–354. 10.1107/S1744309113002674 23545636 PMC3614155

[B62] Tang X. Shao Y. L. Tang Y. J. Zhou W. W. (2018). Antifungal activity of essential oil compounds (geraniol and citral) and inhibitory mechanisms on grain pathogens (*Aspergillus flavus* and *Aspergillus ochraceus*). *Molecules* 23:2108. 10.3390/molecules23092108 30131466 PMC6225121

[B63] Tian J. Wang Y. Zeng H. Li Z. Zhang P. Tessema A. (2015). Efficacy and possible mechanisms of perillaldehyde in control of *Aspergillus niger* causing grape decay. *Int. J. Microbiol.* 202 27–34. 10.1016/j.ijfoodmicro.2015.02.022 25755082

[B64] Valenzuela-Cota D. F. Buitimea-Cantúa G. V. Plascencia-Jatomea M. Cinco-Moroyoqui F. J. Martínez-Higuera A. A. Rosas-Burgos E. C. (2019). Inhibition of the antioxidant activity of catalase and superoxide dismutase from *Fusarium verticillioides* exposed to a *Jacquinia macrocarpa* antifungal fraction. *J. Environ. Sci. Health Part B* 54 647–654. 10.1080/03601234.2019.1622978 31146638

[B65] Vallejo I. Carbú M. Munõz F. Rebordinos L. Cantoral J. M. (2002). Inheritance of chromosome-length polymorphisms in the phytopathogenic ascomycete *Botryotinia fuckeliana* (anam. *Botrytis cinerea*). *Mycol. Res.* 106 1075–1085.

[B66] Vasilaki A. T. McMillan D. C. (2011). “Lipid peroxidation,” in *the Encyclopedia of cancer*, ed. Schwab M. (Berlin: Springer), 2054–2055.

[B67] Velikova V. Yordanov I. Edreva A. (2000). Oxidative stress and some antioxidant systems in acid rain treated bean plants: Protective role of exogenous polyamines. *Plant Sci.* 151 59–66.

[B68] Walker C. L. Pomatto L. Tripathi D. N. Davies K. (2018). Redox regulation of homeostasis and proteostasis in peroxisomes. *Physiol. Rev.* 98 89–115. 29167332 10.1152/physrev.00033.2016 PMC6335096

[B69] Wang W. Vignani R. Scali M. Cresti M. (2006). A universal and rapid protocol for protein extraction from recalcitrant plant tissues for proteomic analysis. *Electrophores* 27 2782–2786. 10.1002/elps.200500722 16732618

[B70] Wilk P. Wątor E. Weiss M. S. (2021). Prolidase – A protein with many faces. *Biochimie.* 183 3–12. 10.1016/j.biochi.2020.09.017 33045291

[B71] Wu Y.-X. Zhang Y.-D. Li N. Wu D.-D. Li Q.-M. Chen Y.-Z. (2022). Inhibitory effect and mechanism of action of juniper essential oil on gray mold in cherry tomatoes. *Front. Microbiol.* 13:1000526. 10.3389/fmicb.2022.1000526 36212845 PMC9537556

[B72] Xenarios I. Eisenberg D. (2001). Protein interaction databases. *Curr. Opin. Biotechnol.* 12 334–339. 11551460 10.1016/s0958-1669(00)00224-x

[B73] Xiong L. Zhu J. K. (2002). Molecular and genetic aspects of plant responses to osmotic stress. *Plant Cell Environ.* 25 131–139. 11841658 10.1046/j.1365-3040.2002.00782.x

[B74] Yakura M. Ishikura Y. Adachi Y. Kawamukai M. (2006). Involvement of Moc1 in sexual development and survival of *Schizosaccharomyces pombe*. *Biosci. Biotechnol. Biochem.* 70 1740–1749. 10.1271/bbb.60088 16819157

[B75] Yang Q. Wang J. Zhang P. Xie S. Yuan X. Hou X. (2020a). In vitro and in vivo antifungal activity and preliminary mechanism of cembratrien-diols against *Botrytis cinerea*. *Indus. Crops Prod.* 154:112745. 10.1016/j.indcrop.2020.112745

[B76] Yang S. K. Tan N. P. Chong C. W. Abushelaibi A. Lim S. H. Lai K. S. (2021). The missing piece: Recent approaches investigating the antimicrobial mode of action of essential oils. *Evol. Bioinform.* 17:1176934320938391. 10.1177/1176934320938391 34017165 PMC8114247

[B77] Yang S. K. Yusoff K. Thomas W. Akseer R. Alhosani M. S. Abushelaibi A. (2020b). Lavender essential oil induces oxidative stress which modifies the bacterial membrane permeability of carbapenemase producing *Klebsiella pneumoniae*. *Sci. Rep.* 10 1–14. 10.1038/s41598-019-55601-0 31964900 PMC6972767

[B78] Yaseen T. Ricelli A. Albanese P. Carboni C. D’Onghia A. M. (2014). “Effect of storage in ozone-enriched atmosphere on fungal contamination, catalase, superoxide dismutase and glutathione peroxidase activity in berries,” in *IOA-EA3G Conference*, (Dublin), 3–5.

[B79] Yasmeen B. Anna K. Craig A. T. (2016). Acute lung injury: A clinical and molecular review. *Arch. Pathol. Lab. Med.* 140 345–350. 10.5858/arpa.2015-0519-ra 27028393

[B80] Zaprasis A. Brill J. Thüring M. Wünsche G. Heun M. Barzantny H. (2013). Osmoprotection of Bacillus subtilis through import and proteolysis of proline-containing peptides. *Appl. Environ. Microbiol.* 79 576–587. 10.1128/AEM.01934-12 23144141 PMC3553765

[B81] Zybailov B. Rutschow H. Friso G. Rudella A. Emanuelsson O. Sun Q. (2008). Sorting signals, N-terminal modifications, and abundance of the chloroplast proteome. *PLoS One* 3:e1994. 10.1371/journal.pone.0001994 18431481 PMC2291561

