# Supplementary material for: Enzymatic and proteomic exploration into the inhibitory activities of lemongrass and lemon essential oils against Botrytis cinerea (causative pathogen of gray mold)
Source: Front Microbiol. 2023 Jan 18;13:1101539. doi: 10.3389/fmicb.2022.1101539 (PMC9890175; doi:10.3389/fmicb.2022.1101539)
Supplement: Supplementary file 1 [file Table_1.DOCX]

***Supplementary (S) -Tables***

**Table S1** List of buffers and stock solutions

| Buffer / Stock solution | | Composition |
| --- | --- | --- |
| Acetone (80%) | 80% (v/v) acetone in d.H_2_O. | |
| Acetone (80%) / 5 mM DTT | 80% (v/v) acetone in 5 mM DTT. | |
| Agarose sealing solution (0.5%) | 0.5% (w/v) agarose prepared in 1 X SDS-PAGE running buffer with a tint of bromophenol blue. | |
| Antioxidant enzyme extraction buffer | 40 mM K_2_HPO_4_ (pH 7.4); 5% polyvinylpyrrolidone (PVP); 5% glycerol; 1 mM EDTA. | |
| APX extraction buffer | 50 mM K_2_HPO_4_ at pH 7.0; 0.2 mM EDTA; buffer 0.25 mM ascorbic acid in dH_2_O. | |
| De-staining solution | 10% (v/v) acetic acid and 1% (v/v) glycerol in d.H_2_O. | |
| DTT (5 mM) | 5 mM in d.H_2_O. | |
| Ethanol (70%) | 70% (v/v) ethanol in d.H_2_O. | |
| HCl (1 M) for pH | 1 M HCl in d.H_2_O. | |
| IEF buffer | 7 M Urea; 2 M Thiourea; 4% (w/v) CHAPS; 20 mM DTT; 1% (w/v) bromophenol blue in d.H_2_O. | |
| Methanol (80%) | 80% methanol in d.H_2_O. | |
| PMSF (100mM) / 100% Isopropanol | 100mM in 100% Isopropanol. | |
| PVP extraction buffer | 40 mM K_2_HPO4 at pH 7.4; 1 mM EDTA; 5% PVP MW = 40 000; 5% glycerol in d.H_2_O. | |
| SDS buffer | 0.1 M Tris-HCl, pH 8.8; 2% (w/v) SDS; 5% (v/v) β-mercaptoethanol; 30% (w/v) sucrose and 1 mM PMSF in d.H_2_O. | |
| SDS gel loading dye | 100 mM Tris-HCl at pH 6.8; 4% (w/v) SDS; 0.2% (w/v) bromophenol blue; 20% (v/v) glycerol; 200 mM DTT in d.H_2_O. | |
| SDS running buffer stock solution (5 X) | 25 mM Tris-base; 192 mM glycine; 0.1% (w/v) SDS in d.H_2_O. | |
| SDS (10%) stock solution | 10% (w/v) SDS in d.H_2_O. | |
| SOD reaction buffer | 50 mM KPO_4_ (pH 7.4); 13 mM L-methionine; 75 μM NBT; 0.1 mM EDTA. | |
| TCA/Acetone (10%) | 10% (w/v) TCA in acetone. | |

**Table S2.** List of all proteins identified from different treatments.

| \| Entry \| Entry name \| GO (cellular component) \| GO (molecular function) \| Protein names \| GO (biological process) \| Organism(s) \| \| --- \| --- \| --- \| --- \| --- \| --- \| --- \| \| O13419 \| ACT_BOTFU \| cytoplasm [GO:0005737]; cytoskeleton [GO:0005856] \| ATP binding [GO:0005524] \| Actin \|  \| *Botryotinia fuckeliana* (Noble rot fungus) (*Botrytis cinerea*) \| \| P22131 \| ACT1_PHYIN \| cytoplasm [GO:0005737]; cytoskeleton [GO:0005856] \| ATP binding [GO:0005524] \| Actin-1 \|  \| *Phytophthora infestans* (Potato late blight agent) (*Botrytis infestans*) \| \| P22132 \| ACT2_PHYIN \| cytoplasm [GO:0005737]; cytoskeleton [GO:0005856] \| ATP binding [GO:0005524] \| Actin-2 \|  \| *Phytophthora infestans* (Potato late blight agent) (*Botrytis infestans*) \| \| P53373 \| TBB_BOTFU \| cytoplasm [GO:0005737]; microtubule [GO:0005874] \| GTP binding [GO:0005525]; structural constituent of cytoskeleton [GO:0005200] \| Tubulin beta chain (Beta-tubulin) \| microtubule-based process [GO:0007017]; response to antibiotic [GO:0046677] \| *Botryotinia fuckeliana* (Noble rot fungus) (*Botrytis cinerea*) \| \| A6S6V7 \| CYNS_BOTFB \|  \| cyanate hydratase activity [GO:0008824]; DNA binding [GO:0003677] \| Cyanate hydratase (Cyanase) (EC 4.2.1.104) (Cyanate hydrolase) (Cyanate lyase) \| cyanate metabolic process [GO:0009439] \| *Botryotinia fuckeliana* (strain B05.10) (Noble rot fungus) (*Botrytis cinerea*) \| \| A7F1L5 \| CYNS_SCLS1 \|  \| cyanate hydratase activity [GO:0008824]; DNA binding [GO:0003677] \| Cyanate hydratase (Cyanase) (EC 4.2.1.104) (Cyanate hydrolase) (Cyanate lyase) \| cyanate metabolic process [GO:0009439] \| *Sclerotinia sclerotiorum* (strain ATCC 18683/1980/Ss-1) (White mold) (*W. sclerotiorum*) \| \| A6RJ45 \| IF4A_BOTFB \| cytoplasm [GO:0005737] \| ATP binding [GO:0005524]; ATP hydrolysis activity [GO:0140603]; RNA helicase activity [GO:0003724]; translation initiation factor activity [GO:0003743] \| ATP-dependent RNA helicase eIF4A (EC 3.6.4.13) \| (Eukaryotic initiation factor 4A) (eIF-4A) (Translation initiation factor 1) \| *Botryotinia fuckeliana* (strain B05.10) (Noble rot fungus) (*Botrytis cinerea*) \| \| A6RPU0 \| KAD2_BOTFB \| cytosol [GO:0005829]; mitochondrial intermembrane space [GO:0005758] \| adenylate kinase activity [GO:0004017]; ATP binding [GO:0005524] \| Adenylate kinase (EC 2.7.4.3) (ATP-AMP transphosphorylase) (ATP:AMP phosphotransferase) (Adenylate kinase cytosolic and mitochondrial) (Adenylate monophosphate kinase) \| ADP biosynthetic process [GO:0006172]; AMP metabolic process [GO:0046033]; ATP metabolic process [GO:0046034] \| *Botryotinia fuckeliana* (strain B05.10) (Noble rot fungus) (*Botrytis cinerea*) \|   **Table S2.** List of all proteins identified from different treatments.   \| Entry \| Entry name \| GO (cellular component) \| GO (molecular function) \| \| Protein names \| \| GO (biological process) \| \| Organism(s) \| \| \| --- \| --- \| --- \| --- \| --- \| --- \| --- \| --- \| --- \| --- \| --- \| \| A7E8H8 \| KAD2_SCLS1 \| cytoplasm [GO:0005737]; cytosol [GO:0005829]; mitochondrial intermembrane space [GO:0005758]; mitochondrion [GO:0005739] \| adenylate kinase activity [GO:0004017]; ATP binding [GO:0005524] \| \| Adenylate kinase (EC 2.7.4.3) (ATP-AMP transphosphorylase) (ATP:AMP phosphotransferase) (Adenylate kinase cytosolic and mitochondrial) (Adenylate monophosphate kinase) \| \| ADP biosynthetic process [GO:0006172]; AMP metabolic process [GO:0046033]; ATP metabolic process [GO:0046034] \| \| *Sclerotinia sclerotiorum* (strain ATCC 18683 / 1980 / Ss-1) (White mold) (*Whetzelinia sclerotiorum*) \| \| \| A6RPN7 \| BGALB_BOTFB \| extracellular region [GO:0005576] \| beta-galactosidase activity [GO:0004565] \| \| Probable beta-galactosidase B (EC 3.2.1.23) (Lactase B) \| \| polysaccharide catabolic process [GO:0000272] \| \| *Botryotinia fuckeliana* (strain B05.10) (Noble rot fungus) (*B. cinerea*) \| \| \| A7EBU5 \| BGALB_SCLS1 \| cell wall [GO:0005618]; extracellular region [GO:0005576]; vacuole [GO:0005773] \| beta-galactosidase activity [GO:0004565] \| \| Probable beta-galactosidase B (EC 3.2.1.23) (Lactase B) \| \| polysaccharide catabolic process [GO:0000272] \| \| *Sclerotinia sclerotiorum* (strain ATCC 18683 / 1980 / Ss-1) (White mold) (*W. sclerotiorum*) \| \| \| A6SEH9 \| DED1_BOTFB \| cytoplasm [GO:0005737] \| \| ATP binding [GO:0005524]; ATP hydrolysis activity [GO:0140603]; RNA helicase activity [GO:0003724]; translation initiation factor activity [GO:0003743] \| \| ATP-dependent RNA helicase ded1 (EC 3.6.4.13) \| \|  \| \| *Botryotinia fuckeliana* (strain B05.10) (Noble rot fungus) *(Botrytis cinerea*) \| \| A7F2R3 \| RSSA_SCLS1 \| cytosolic small ribosomal subunit [GO:0022627] \| \| structural constituent of ribosome [GO:0003735] \| \| 40S ribosomal protein S0 \| \| cytoplasmic translation [GO:0002181]; ribosomal small subunit assembly [GO:0000028]; translation [GO:0006412] \| \| *Sclerotinia sclerotiorum* (strain ATCC 18683 / 1980 / Ss-1) (White mold) (*Whetzelinia sclerotiorum*) \| \| A6S8A1 \| PFF1_BOTFB \| integral component of membrane [GO:0016021]; vacuolar membrane [GO:0005774] \| \| metal ion binding [GO:0046872]; metalloexopeptidase activity [GO:0008235] \| \| Vacuolar membrane protease (EC 3.4.-.-) (FXNA-related family protease 1) \| \|  \| \| *Botryotinia fuckeliana* (strain B05.10) (Noble rot fungus) (*Botrytis cinerea*) \| \| A7F6C1 \| MIC60_SCLS1 \| MICOS complex [GO:0061617] \| \|  \| \| MICOS complex subunit mic60 (Mitofilin) \| \| cristae formation [GO:0042407] \| \| *S. sclerotiorum* (strain ATCC 18683 / 1980 / Ss-1) (White mold) (*W. sclerotiorum*) \| \| A7ENU3 \| CLU_SCLS1 \| cytoplasm [GO:0005737] \| \| mRNA binding [GO:0003729] \| \| Clustered mitochondria protein homolog (Protein TIF31 homolog) \| \| intracellular distribution of mitochondria [GO:0048312] \| \| *S. sclerotiorum* (strain ATCC 18683 / 1980 / Ss-1) (White mold) (*W. sclerotiorum*) \|   **Table S2.** List of all proteins identified from different treatments.   \| Entry \| Entry name \| GO (cellular component) \| GO (molecular function) \| Protein names \| GO (biological process) \| Organism(s) \| \| --- \| --- \| --- \| --- \| --- \| --- \| --- \| \| A6SI59 \| MCR1_BOTFB \| integral component of membrane [GO:0016021]; mitochondrial outer membrane [GO:0005741] \| cytochrome-b5 reductase activity, acting on NAD(P)H [GO:0004128] \| NADH-cytochrome b5 reductase 2 (EC 1.6.2.2) (Mitochondrial cytochrome b reductase) \|  \| *Botryotinia fuckeliana* (strain B05.10) (Noble rot fungus) (*Botrytis cinerea*) \| \| P87018 \| RAS_BOTFU \| plasma membrane [GO:0005886] \| G protein activity [GO:0003925]; GTP binding [GO:0005525] \| Ras-like protein (EC 3.6.5.2) \| signal transduction [GO:0007165] \| *Botryotinia fuckeliana* (Noble rot fungus) (*Botrytis cinerea*) \| \| Q86ZC1 \| KINH_BOTFU \| cytoplasm [GO:0005737]; microtubule [GO:0005874] \| ATP binding [GO:0005524]; ATP-dependent microtubule motor activity [GO:1990939]; microtubule binding [GO:0008017] \| Kinesin heavy chain \| microtubule-based movement [GO:0007018] \| *Botryotinia fuckeliana* (Noble rot fungus) (*Botrytis cinerea*) \| \| O74268 \| H2A_BOTFB \| nucleosome [GO:0000786]; nucleus [GO:0005634] \| DNA binding [GO:0003677]; protein heterodimerization activity [GO:0046982] \| Histone H2A \| DNA repair [GO:0006281] \| *Botryotinia fuckeliana* (strain B05.10) (Noble rot fungus) (*Botrytis cinerea*) \| \| A6SFQ6 \| EIF3B_BOTFB \| eukaryotic 43S preinitiation complex [GO:0016282]; eukaryotic 48S preinitiation complex [GO:0033290]; eukaryotic translation initiation factor 3 complex [GO:0005852] \| translation initiation factor activity [GO:0003743]; translation initiation factor binding [GO:0031369] \| Eukaryotic translation initiation factor 3 subunit B (eIF3b) (Eukaryotic translation initiation factor 3 90 kDa subunit homolog) (eIF3 p90) (Translation initiation factor eIF3, p90 subunit homolog) \| formation of cytoplasmic translation initiation complex [GO:0001732] \| *Botryotinia fuckeliana* (strain B05.10) (Noble rot fungus) (*Botrytis cinerea*) \| \| A7EHM8 \| EIF3B_SCLS1 \| eukaryotic 43S preinitiation complex [GO:0016282]; eukaryotic 48S preinitiation complex [GO:0033290]; eukaryotic translation initiation factor 3 complex [GO:0005852] \| translation initiation factor activity [GO:0003743]; translation initiation factor binding [GO:0031369] \| Eukaryotic translation initiation factor 3 subunit B (eIF3b) (Eukaryotic translation initiation factor 3 90 kDa subunit homolog) (eIF3 p90) (Translation initiation factor eIF3, p90 subunit homolog) \| formation of cytoplasmic translation initiation complex [GO:0001732]; translational initiation [GO:0006413] \| *Sclerotinia sclerotiorum* (strain ATCC 18683 / 1980 / Ss-1) (White mold) (*Whetzelinia sclerotiorum*) \| \| A6RZS5 \| RS3A_BOTFB \| cytosolic small ribosomal subunit [GO:0022627] \| structural constituent of ribosome [GO:0003735] \| 40S ribosomal protein S1 \| translation [GO:0006412] \| *Botryotinia fuckeliana* (strain B05.10) (Noble rot fungus) (*B. cinerea*) \|   **S-Table 2.** List of all proteins identified from different treatments.   \| Entry \| Entry name \| GO (cellular component) \| GO (molecular function) \| Protein names \| GO (biological process) \| Organism(s) \| \| --- \| --- \| --- \| --- \| --- \| --- \| --- \| \| A6S146 \| ARGJ1_BOTFB \| mitochondrial matrix [GO:0005759] \| acetyl-CoA:L-glutamate N-acetyltransferase activity [GO:0004042]; glutamate N-acetyltransferase activity [GO:0004358]; methione N-acyltransferase activity [GO:0103045] \| Arginine biosynthesis bifunctional protein ArgJ 1, mitochondrial [Cleaved into: Arginine biosynthesis bifunctional protein ArgJ 1 alpha chain; Arginine biosynthesis bifunctional protein ArgJ 1 beta chain] [Includes: Glutamate N-acetyltransferase (GAT) (EC 2.3.1.35) (Ornithine acetyltransferase) (OATase) (Ornithine transacetylase); Amino-acid acetyltransferase (EC 2.3.1.1) (N-acetylglutamate synthase) (AGS)] \| arginine biosynthetic process [GO:0006526] \| *Botryotinia fuckeliana* (strain B05.10) (Noble rot fungus) (*Botrytis cinerea*) \| \| A7E5P6 \| ARGJ1_SCLS1 \| mitochondrial matrix [GO:0005759] \| acetyl-CoA:L-glutamate N-acetyltransferase activity [GO:0004042]; glutamate N-acetyltransferase activity [GO:0004358]; methione N-acyltransferase activity [GO:0103045] \| Arginine biosynthesis bifunctional protein ArgJ 1, mitochondrial [Cleaved into: Arginine biosynthesis bifunctional protein ArgJ 1 alpha chain; Arginine biosynthesis bifunctional protein ArgJ 1 beta chain] [Includes: Glutamate N-acetyltransferase (GAT) (EC 2.3.1.35) (Ornithine acetyltransferase) (OATase) (Ornithine transacetylase); Amino-acid acetyltransferase (EC 2.3.1.1) (N-acetylglutamate synthase) (AGS)] \| arginine biosynthetic process [GO:0006526]; ornithine biosynthetic process [GO:0006592] \| *Sclerotinia sclerotiorum* (strain ATCC 18683 / 1980 / Ss-1) (White mold) (*Whetzelinia sclerotiorum*) \| \| A1IVT7 \| HOG1_BOTFB \| cytoplasm [GO:0005737]; nucleus [GO:0005634] \| ATP binding [GO:0005524]; MAP kinase activity [GO:0004707]; protein serine kinase activity [GO:0106310]; protein threonine kinase activity [GO:0106311] \| Mitogen-activated protein kinase hog1 (MAP kinase hog1) (EC 2.7.11.24) (BcSAK1) (Stress-activated mitogen-activated protein kinase) \| stress-activated MAPK cascade [GO:0051403] \| *Botryotinia fuckeliana* (strain B05.10) (Noble rot fungus) (*Botrytis cinerea*) \| \| A6RY31 \| DHH1_BOTFB \| P-body [GO:0000932] \| ATP binding [GO:0005524]; ATP hydrolysis activity [GO:0140603]; RNA binding [GO:0003723]; RNA helicase activity [GO:0003724] \| ATP-dependent RNA helicase dhh1 (EC 3.6.4.13) \| mRNA processing [GO:0006397]; mRNA transport [GO:0051028]; regulation of translation [GO:0006417] \| *Botryotinia fuckeliana* (strain B05.10) (Noble rot fungus) (*Botrytis cinerea*) \|   **S-Table 2.** List of all proteins identified from different treatments.   \| Entry \| Entry name \| GO (cellular component) \| \| GO (molecular function) \| Protein names \| GO (biological process) \| Organism(s) \| \| --- \| --- \| --- \| --- \| --- \| --- \| --- \| --- \| \| A7E8B6 \| SLA1_SCLS1 \| actin cortical patch [GO:0030479]; endosome membrane [GO:0010008]; nucleus [GO:0005634]; plasma membrane [GO:0005886] \| \| actin binding [GO:0003779]; identical protein binding [GO:0042802]; protein-macromolecule adaptor activity [GO:0030674]; ubiquitin binding [GO:0043130] \| Actin cytoskeleton-regulatory complex protein sla1 \| actin cortical patch assembly [GO:0000147]; actin cortical patch organization [GO:0044396]; endocytosis [GO:0006897]; endosomal transport [GO:0016197] \| *Sclerotinia sclerotiorum* (strain ATCC 18683 / 1980 / Ss-1) (White mold) (*Whetzelinia sclerotiorum*) \| \| A6SFW7 \| DBP2_BOTFB \| cytoplasm [GO:0005737]; nucleus [GO:0005634] \| \| ATP binding [GO:0005524]; ATP hydrolysis activity [GO:0140603]; RNA binding [GO:0003723]; RNA helicase activity [GO:0003724] \| ATP-dependent RNA helicase dbp2 (EC 3.6.4.13) \| nuclear-transcribed mRNA catabolic process, nonsense-mediated decay [GO:0000184]; rRNA processing [GO:0006364] \| *Botryotinia fuckeliana* (strain B05.10) (Noble rot fungus) (*Botrytis cinerea*) \| \| A6S7T2 \| GET3_BOTFB \| endoplasmic reticulum [GO:0005783] \| \| ATP binding [GO:0005524]; hydrolase activity [GO:0016787]; metal ion binding [GO:0046872] \| ATPase get3 (EC 3.6.-.-) (Arsenical pump-driving ATPase) (Arsenite-stimulated ATPase) (Golgi to ER traffic protein 3) (Guided entry of tail-anchored proteins 3) \| protein insertion into ER membrane [GO:0045048] \| *Botryotinia fuckeliana* (strain B05.10) (Noble rot fungus) (*B. cinerea*) \| \| A6SDE9 \| AMPP3_BOTFB \| \|  \| manganese ion binding [GO:0030145]; metalloaminopeptidase activity [GO:0070006] \| Probable Xaa-Pro aminopeptidase pepP (EC 3.4.11.9) (Aminoacylproline aminopeptidase) (Prolidase) \|  \| *Botryotinia fuckeliana* (strain B05.10) (Noble rot fungus) (*B. cinerea*) \| \| A6SJW6 \| EIF3D_BOTFB \| eukaryotic 43S preinitiation complex [GO:0016282]; eukaryotic 48S preinitiation complex [GO:0033290]; eukaryotic translation initiation factor 3 complex [GO:0005852] \| \| mRNA cap binding [GO:0098808]; translation initiation factor activity [GO:0003743] \| Eukaryotic translation initiation factor 3 subunit D (eIF3d) \| cap-dependent translational initiation [GO:0002191]; formation of cytoplasmic translation initiation complex [GO:0001732] \| *Botryotinia fuckeliana* (strain B05.10) (Noble rot fungus) (*Botrytis cinerea*) \| \| A6SBT4 \| DBP5_BOTFB \| cytoplasm [GO:0005737]; nuclear membrane [GO:0031965]; nuclear pore [GO:0005643] \| \| ATP binding [GO:0005524]; ATP hydrolysis activity [GO:0140603]; RNA binding [GO:0003723]; RNA helicase activity [GO:0003724] \| ATP-dependent RNA helicase dbp5 (EC 3.6.4.13) \| mRNA transport [GO:0051028]; protein transport [GO:0015031] \| *Botryotinia fuckeliana* (strain B05.10) (Noble rot fungus) (*Botrytis cinerea*) \|   **S-Table 2.** List of all proteins identified from different treatments.   \| Entry \| Entry name \| \| GO (cellular component) \| \| GO (molecular function) \| \| Protein names \| \| GO (biological process) \| Organism(s) \| \| --- \| --- \| --- \| --- \| --- \| --- \| --- \| --- \| --- \| --- \| --- \| \| A7EIX7 \| SUB2_SCLS1 \| \| spliceosomal complex [GO:0005681] \| \| ATP binding [GO:0005524]; ATP hydrolysis activity [GO:0140603]; RNA binding [GO:0003723]; RNA helicase activity [GO:0003724] \| \| ATP-dependent RNA helicase sub2 (EC 3.6.4.13) \| \| mRNA export from nucleus [GO:0006406]; mRNA splicing, via spliceosome [GO:0000398] \| *Sclerotinia sclerotiorum* (strain ATCC 18683 / 1980 / Ss-1) (White mold) (*W. sclerotiorum*) \| \| A6SL49 \| DAPB_BOTFB \| \| integral component of membrane [GO:0016021]; vacuolar membrane [GO:0005774] \| \| aminopeptidase activity [GO:0004177]; dipeptidyl-peptidase activity [GO:0008239]; serine-type peptidase activity [GO:0008236] \| \| Probable dipeptidyl-aminopeptidase B (DPAP B) (EC 3.4.14.5) \| \|  \| *Botryotinia fuckeliana* (strain B05.10) (Noble rot fungus) (*B. cinerea*) \| \| A6S1A3 \| \| EIF3L_BOTFB \| \| eukaryotic 43S preinitiation complex [GO:0016282]; eukaryotic 48S preinitiation complex [GO:0033290]; eukaryotic translation initiation factor 3 complex [GO:0005852] \| translation initiation factor activity [GO:0003743] \| Eukaryotic translation initiation factor 3 subunit L (eIF3l) \| \| formation of cytoplasmic translation initiation complex [GO:0001732] \| \| *Botryotinia fuckeliana* (strain B05.10) (Noble rot fungus) (*Botrytis cinerea*) \| \| Q2LMP0 \| \| XY11A_BOTFU \| \| extracellular region [GO:0005576] \| endo-1,4-beta-xylanase activity [GO:0031176] \| Endo-1,4-beta-xylanase 11A (Xylanase 11A) (EC 3.2.1.8) (1,4-beta-D-xylan xylanohydrolase 11A) \| \| xylan catabolic process [GO:0045493] \| \| *Botryotinia fuckeliana* (Noble rot fungus) (*Botrytis cinerea*) \| \| Q70Q35 \| \| SODC_BOTFU \| \| cytoplasm [GO:0005737] \| metal ion binding [GO:0046872]; superoxide dismutase activity [GO:0004784] \| Superoxide dismutase [Cu-Zn] (EC 1.15.1.1) \| \|  \| \| *Botryotinia fuckeliana* (Noble rot fungus) (*B. cinerea*) \| \| A7F3V4 \| \| SDS23_SCLS1 \| \| cytoplasm [GO:0005737]; nucleotide-activated protein kinase complex [GO:0031588]; nucleus [GO:0005634] \| AMP binding [GO:0016208]; protein kinase binding [GO:0019901]; protein kinase regulator activity [GO:0019887]; protein serine/threonine phosphatase inhibitor activity [GO:0004865] \| Protein sds23 \| \| cellular response to glucose starvation [GO:0042149]; protein phosphorylation [GO:0006468]; regulation of catalytic activity [GO:0050790]; regulation of mitotic metaphase/anaphase transition [GO:0030071] \| \| *Sclerotinia sclerotiorum* (strain ATCC 18683 / 1980 / Ss-1) (White mold) (*Whetzelinia sclerotiorum*) \|   **S-Table 2.** List of all proteins identified from different treatments.   \| Entry \| Entry name \| GO (cellular component) \| \| GO (molecular function) \| Protein names \| GO (biological process) \| Organism(s) \| \| --- \| --- \| --- \| --- \| --- \| --- \| --- \| --- \| \| A6S544 \| SEY1_BOTFB \| endoplasmic reticulum membrane [GO:0005789]; integral component of membrane [GO:0016021] \| \| GTPase activity [GO:0003924]; GTP binding [GO:0005525] \| Protein sey1 (EC 3.6.5.) \| endoplasmic reticulum organization [GO:0007029] \| *Botryotinia fuckeliana* (strain B05.10) (Noble rot fungus) (*Botrytis cinerea*) \| \| A7ERA6 \| SEY1_SCLS1 \| endoplasmic reticulum [GO:0005783]; endoplasmic reticulum membrane [GO:0005789]; integral component of membrane [GO:0016021] \| \| GTPase activity [GO:0003924]; GTP binding [GO:0005525] \| Protein sey1 (EC 3.6.5.) \| endoplasmic reticulum membrane fusion [GO:0016320] \| *Sclerotinia sclerotiorum* (strain ATCC 18683/1980/Ss-1) (White mold) (*Whetzelinia sclerotiorum*) \| \| A6SB28 \| NACA_BOTFB \| nascent polypeptide-associated complex [GO:0005854]; nucleus [GO:0005634] \|  \| \| Nascent polypeptide-associated complex subunit alpha (NAC-alpha) (Alpha-NAC) \| protein transport [GO:0015031] \| *Botryotinia fuckeliana* (strain B05.10) (Noble rot fungus) (*Botrytis cinerea*) \| \| P55304 \| CATA_BOTFU \| peroxisome [GO:0005777] \| \| catalase activity [GO:0004096]; heme binding [GO:0020037]; metal ion binding [GO:0046872] \| Catalase A (EC 1.11.1.6) \| hydrogen peroxide catabolic process [GO:0042744]; response to oxidative stress [GO:0006979] \| *Botryotinia fuckeliana* (Noble rot fungus) (*Botrytis cinerea*) \| \| A6RUL1 \| EIF3I_BOTFB \| eukaryotic 43S preinitiation complex [GO:0016282]; eukaryotic 48S preinitiation complex [GO:0033290]; eukaryotic translation initiation factor 3 complex [GO:0005852] \| \| translation initiation factor activity [GO:0003743] \| Eukaryotic translation initiation factor 3 subunit I (eIF3i) (Eukaryotic translation initiation factor 3 39 kDa subunit homolog) (eIF-3 39 kDa subunit homolog) \| formation of cytoplasmic translation initiation complex [GO:0001732] \| *Botryotinia fuckeliana* (strain B05.10) (Noble rot fungus) (*Botrytis cinerea*) \| \| A7F9Y3 \| S2538_SCLS1 \| integral component of membrane [GO:0016021]; mitochondrial inner membrane [GO:0005743]; mitochondrion [GO:0005739] \| \| Glycine transmembrane transporter activity [GO:0015187] \| Mitochondrial glycine transporter (Solute carrier family 25-member 38 homolog) \| glycine imports into mitochondrion [GO:1904983] \| *Sclerotinia sclerotiorum* (strain ATCC 18683 / 1980 / Ss-1) (White mold) (*Whetzelinia sclerotiorum*) \|   **S-Table 2.** List of all proteins identified from different treatments.   \| Entry \| Entry name \| GO (cellular component) \| \| GO (molecular function) \| \| Protein names \| GO (biological process) \| Organism \| \| --- \| --- \| --- \| --- \| --- \| --- \| --- \| --- \| --- \| \| A7ETB3 \| MDV1_SCLS1 \| mitochondrial outer membrane [GO:0005741]; mitochondrion [GO:0005739]; pre-ribosome, large subunit precursor [GO:0030687] \| \| \|  \| Mitochondrial division protein 1 \| mitochondrial fission [GO:0000266]; peroxisome fission [GO:0016559] \| *Sclerotinia sclerotiorum* (strain ATCC 18683 / 1980 / Ss-1) (White mold) (*Whetzelinia sclerotiorum*) \| \| M7UQV4 \| ATG3_BOTF1 \| cytoplasm [GO:0005737] \|  \| \| \| Autophagy-related protein 3 (Autophagy-related E2-like conjugation enzyme atg3) \| autophagy [GO:0006914]; protein transport [GO:0015031] \| *Botryotinia fuckeliana* (strain BcDW1) (Noble rot fungus) (*Botrytis cinerea*) \| \| A6RMY5 \| NOP58_BOTFB \| nucleolus [GO:0005730] \|  \| \| \| Nucleolar protein 58 \| rRNA processing [GO:0006364] \| *Botryotinia fuckeliana* (strain B05.10) (Noble rot fungus) (*B. cinerea*) \| \| A6SM77 \| EIF3E_BOTFB \| eukaryotic 43S preinitiation complex [GO:0016282]; eukaryotic 48S preinitiation complex [GO:0033290]; eukaryotic translation initiation factor 3 complex, eIF3e [GO:0071540] \| translation initiation factor activity [GO:0003743] \| \| \| Eukaryotic translation initiation factor 3 subunit E (eIF3e) \| formation of cytoplasmic translation initiation complex [GO:0001732] \| *Botryotinia fuckeliana* (strain B05.10) (Noble rot fungus) (*B. cinerea*) \| \| A6S3W1 \| ROT1_BOTFB \| endoplasmic reticulum membrane [GO:0005789]; integral component of membrane [GO:0016021] \| Protein rot1 \| \| \| \| 'de novo' protein folding [GO:0006458] \| *Botryotinia fuckeliana* (strain B05.10) (Noble rot fungus) (*B. cinerea*) \| \| P0CV60 \| RL144_PLAVT \| extracellular region [GO:0005576]; host cell cytoplasm [GO:0030430]; host cell nucleus [GO:0042025] \| \| \| \| Secreted RxLR effector protein 144 \|  \| *Plasmopara viticola* (Downy mildew of grapevine) (*B. viticola*) \| |
| --- | --- | --- | --- | --- | --- | --- | --- | --- | --- | --- | --- | --- | --- | --- | --- | --- | --- | --- | --- | --- | --- | --- | --- | --- | --- | --- | --- | --- | --- | --- | --- | --- | --- | --- | --- | --- | --- | --- | --- | --- | --- | --- | --- | --- | --- | --- | --- | --- | --- | --- | --- | --- | --- | --- | --- | --- | --- | --- | --- | --- | --- | --- | --- | --- | --- | --- | --- | --- | --- | --- | --- | --- | --- | --- | --- | --- | --- | --- | --- | --- | --- | --- | --- | --- | --- | --- | --- | --- | --- | --- | --- | --- | --- | --- | --- | --- | --- | --- | --- | --- | --- | --- | --- | --- | --- | --- | --- | --- | --- | --- | --- | --- | --- | --- | --- | --- | --- | --- | --- | --- | --- | --- | --- | --- | --- | --- | --- | --- | --- | --- | --- | --- | --- | --- | --- | --- | --- | --- | --- | --- | --- | --- | --- | --- | --- | --- | --- | --- | --- | --- | --- | --- | --- | --- | --- | --- | --- | --- | --- | --- | --- | --- | --- | --- | --- | --- | --- | --- | --- | --- | --- | --- | --- | --- | --- | --- | --- | --- | --- | --- | --- | --- | --- | --- | --- | --- | --- | --- | --- | --- | --- | --- | --- | --- | --- | --- | --- | --- | --- | --- | --- | --- | --- | --- | --- | --- | --- | --- | --- | --- | --- | --- | --- | --- | --- | --- | --- | --- | --- | --- | --- | --- | --- | --- | --- | --- | --- | --- | --- | --- | --- | --- | --- | --- | --- | --- | --- | --- | --- | --- | --- | --- | --- | --- | --- | --- | --- | --- | --- | --- | --- | --- | --- | --- | --- | --- | --- | --- | --- | --- | --- | --- | --- | --- | --- | --- | --- | --- | --- | --- | --- | --- | --- | --- | --- | --- | --- | --- | --- | --- | --- | --- | --- | --- | --- | --- | --- | --- | --- | --- | --- | --- | --- | --- | --- | --- | --- | --- | --- | --- | --- | --- | --- | --- | --- | --- | --- | --- | --- | --- | --- | --- | --- | --- | --- | --- | --- | --- | --- | --- | --- | --- | --- | --- | --- | --- | --- | --- | --- | --- | --- | --- | --- | --- | --- | --- | --- | --- | --- | --- | --- | --- | --- | --- | --- | --- | --- | --- | --- | --- | --- | --- | --- | --- | --- | --- | --- | --- | --- | --- | --- | --- | --- | --- | --- | --- | --- | --- | --- | --- | --- | --- | --- | --- | --- | --- | --- | --- | --- | --- | --- | --- | --- | --- | --- | --- | --- | --- | --- | --- | --- | --- | --- | --- | --- | --- | --- | --- | --- | --- | --- | --- | --- | --- | --- | --- | --- | --- | --- | --- | --- | --- | --- | --- | --- | --- | --- | --- | --- | --- | --- | --- | --- | --- | --- | --- | --- | --- | --- | --- | --- | --- | --- | --- | --- | --- | --- | --- | --- | --- | --- | --- | --- | --- | --- | --- | --- | --- | --- | --- | --- | --- | --- | --- | --- | --- | --- | --- | --- | --- | --- | --- | --- | --- | --- | --- | --- | --- | --- | --- | --- | --- | --- | --- | --- | --- | --- | --- | --- | --- | --- | --- | --- | --- | --- | --- | --- | --- | --- | --- | --- | --- | --- | --- | --- | --- | --- | --- | --- | --- | --- | --- | --- | --- | --- |

## Supplementary Figures


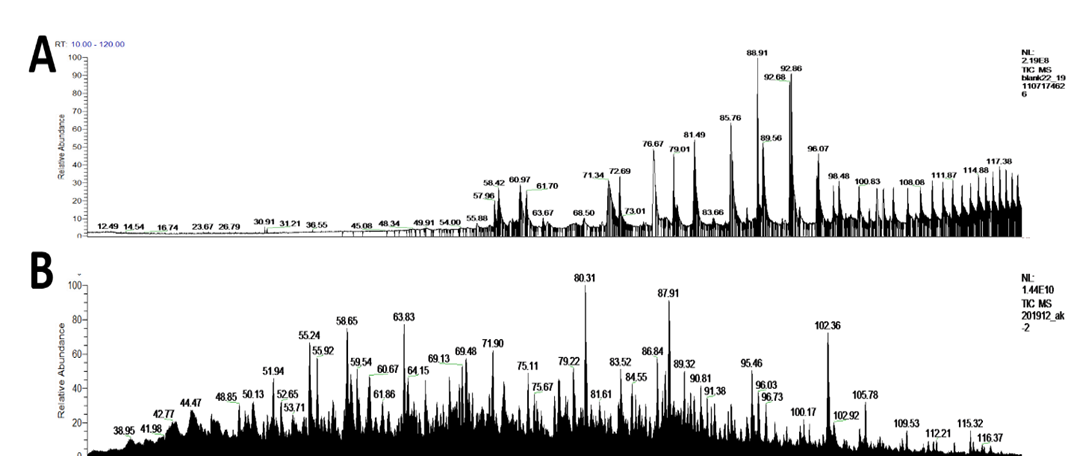


**Figure S1.** Total ion chromatograms of the system blank (A) and suitability mixture (B) injection.


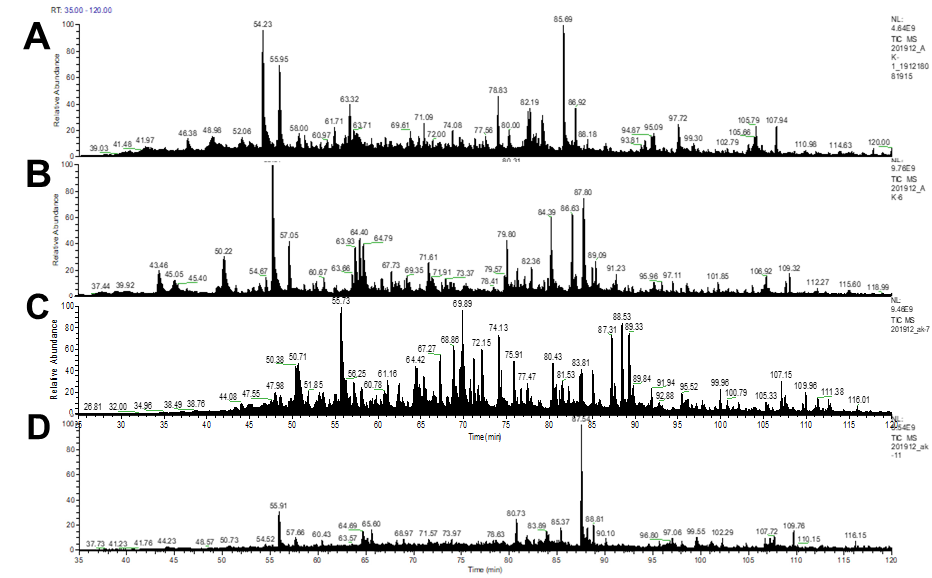


**Figure S2.** The total ion chromatography LC-MS analysis of *B. cinerea* treated with essential oil. (A) Control; (B) Le; (C) Lg and (D) Le + Lg EOs.
